# Heterogeneity in Family Life Course Patterns and Intra-Cohort Wealth Disparities in Late Working Age

**DOI:** 10.1007/s10680-021-09601-4

**Published:** 2021-12-23

**Authors:** Nicole Kapelle, Sergi Vidal

**Affiliations:** 1 Department of Sociology, University of Oxford, 42-43 Park End Street, Oxford, OX1 1JD England; 2 Nuffield College, University of Oxford, New Road, Oxford, OX1 1NF England; 3 Leverhulme Centre for Demographic Science (LCDS), University of Oxford, 42-43 Park End Street, Oxford, OX1 1JD England; 4 Centre d’Estudis Demogràfics, Universitat Autònoma de Barcelona, Carrer de Ca n’Altayó, Edifici E2, 08193 Bellaterra/Barcelona, Spain

**Keywords:** Family, Life course, Inequality/Social stratification, Wealth, Gender

## Abstract

Considering soaring wealth inequalities in older age, this research addresses the relationship between family life courses and widening wealth differences between individuals as they age. We holistically examine how childbearing and marital histories are associated with personal wealth at ages 50–59 for Western Germans born between 1943 and 1967. We propose that deviations from culturally and institutionally-supported family patterns, or the stratified access to them, associate with differential wealth accumulation over time and can explain wealth inequalities at older ages. Using longitudinal data from the German Socio-Economic Panel Study (SOEP, v34, waves 2002–2017), we first identified typical family trajectory patterns between ages 16 and 50 with multichannel sequence analysis and cluster analysis. We then modelled personal wealth ranks at ages 50–59 as a function of family patterns. Results showed that deviations from the standard family pattern (i.e. stable marriage with, on average, two children) were mostly associated with lower wealth ranks at older age, controlling for childhood characteristics that partly predict selection into family patterns and baseline wealth. We found higher wealth penalties for greater deviation and lower penalties for moderate deviation from the standard family pattern. Addressing entire family trajectories, our research extended and nuanced our knowledge of the role of earlier family behaviour for later economic wellbeing. By using personal-level rather than household-level wealth data, we were able to identify substantial gender differences in the study associations. Our research also recognised the importance of combining marital and childbearing histories to assess wealth inequalities.

**Supplementary Information:**

The online version contains supplementary material available at 10.1007/s10680-021-09601-4.

## Introduction

In the light of an ageing population and its increasing pressure on the welfare system, countries with generous social welfare systems such as Germany have increasingly emphasised personal responsibility and more market-based solutions to ensure economic wellbeing throughout one’s life (Ebbinghaus, 2015; Seeleib-Kaiser, 2016). Personal savings and other private sources of wealth are thus increasingly relevant to the future living standards of the contemporary workforce. However, individuals differ markedly in the rate at which they accumulate wealth over their working lives, which is reflected in recent trends of soaring wealth inequalities in older age in most wealthy nations (OECD, 2013). In the longer term, widening wealth disparities at older ages will increase reliance on welfare, hinder social cohesion, and contribute to rising economic inequality through the unequal intergenerational transmission of resources and opportunities (Pfeffer & Killewald, 2017; Pfeffer & Schoeni, 2016).

When examining potential sources of wealth inequalities, research, and policy has traditionally focused on the role of labour market position and social background (e.g. Atkinson, 1971; Bernardi et al., 2018; Lillard, 1977; Ponomarenko, 2017). In more recent decades, family roles—and transitions across these roles—have additionally been recognised as relevant to socio-economic stratification and wealth inequality (e.g. Halpern-Manners et al., 2015; Hurd, 2002; McLanahan & Percheski, 2008; Zissimopoulos et al., 2015).

The focus on the family is particularly relevant considering demographic developments since the 1950s. Economic growth and mass prosperity in the post-war period were accompanied by the golden age of marriage and standardisation trends in the family domain. A relatively early, stable marriage closely followed by several childbirths developed into the demographically dominant family pattern. Arguably, this post-war “standard” family pattern is not dominant anymore given pervasive trends of de-standardisation and pluralisation of family life courses since the 1960s—characterized by declines in and postponement of marriage and childbearing, as well as by the emergence of new family arrangements such as unmarried couples with children, lone parents or step- and blended families—that resulted in a new structural heterogeneity of contemporary family lives (Brückner & Mayer, 2005; Kohli, 2007; Zimmermann & Konietzka, 2017). However, some key features of post-war family arrangements such as a stable marriage with (on average, two) children are still perceived as the cultural ideal and believed to be economically enhancing. Relatedly, it has been argued that pervasive changes in the family realm have exacerbated socio-economic disadvantages. On one hand, persisting cultural and institutional support for the standard family pattern have meant that substantial economic benefits are associated with its long-term enactment, while following an alternative family pattern was often sanctioned (Lersch, 2017). One the other hand, the increasing salience of economic prerequisites for marriage and family formation have led to stratified access to the standard family pattern that often exclude disadvantaged individuals and social groups who perceive these prerequisites as unachievable (Gibson-Davis et al., 2005). Either way, the increasing heterogeneity in family roles is found to broaden individual differentials in wealth accumulation and can contribute to growing wealth inequality at older ages. Whether and how the latter occurs, however, remains an empirical question.

To close these gaps in our knowledge, the present study examines whether heterogeneity in family life courses from ages 16 to 50 is associated with wealth disparities at age 50 to 59 among cohorts of West Germans born between 1943 and 1967. A consideration of these cohorts is ideal as they are arguably the forerunners of the de-standardisation of family life courses with later study cohorts (i.e. respondents born in the 1960s) displaying less uniformity in family life courses than the earlier study cohorts (e.g. Huinink, 2013). We identify the intra-cohort *heterogeneity* in family trajectories by establishing a typology of trajectory patterns and assess the extent to which deviation from a *standard* family pathway are associated with lower wealth at older ages, as a potential result of breaking with the associated mechanisms of wealth accumulation or due to stratified access to different family pathways. We additionally assess what *type of trajectory patterns* matters and can further help to understand disparities in wealth accumulation at older ages. We consider the extent to which all these associations vary by gender, as wealth accumulation potentials have been shown to differ between men and women (Bessière, 2019; Sierminska et al., 2010).

Due to substantially different economic and welfare systems across the Federal Republic of Germany (FRG) in the West and the German Democratic Republic (GDR) in the East between 1949 and 1990, we solely consider West Germany in the current study.^1^ West Germany provides an interesting case as it has been characterized by persistent cultural and institutional support for traditional family arrangements featuring stable marriage and a male breadwinner model, despite pervasive changes in the societal roles and personal endowments of women, as well as in partnership and fertility behaviours (Trappe et al., 2015).

The present study extends existing research on the association between family life courses and later life wealth in three important ways. First, we adopt a holistic life course approach to assess family life courses as long-term trajectories. Previous wealth research relied on blunt summary indicators of past point-in-time family outcomes (e.g. being ever divorced, currently married, divorced twice) to classify entire family life courses. This approach has obscured the heterogeneity in pathways leading to similar family outcomes but different economic wellbeing in older age (Halpern-Manners et al., 2015). Our approach enables us to explicitly acknowledge that an aggregate of time-dependent processes featuring the occurrences, timings, and ordering of family transitions are (directly or indirectly) related to the life-long accumulation of economic resources and thus contributes to intra-cohort wealth inequality. While similar longitudinal approaches were taken by Madero-Cabib and Fasang (2016), Muller et al. (2020) or Jalovaara and Fasang (2020) to examine the association between family life courses and income in mid- to late-life, it needs to acknowledged that wealth is not a direct function of income, as aspects such as consumption or financial transfers additionally influence wealth accumulation (Killewald et al., 2017).

Second, we examine marital *and* fertility histories simultaneously to assess how the intersection of these two life course domains is linked to wealth in older age. Thus, we acknowledge increasingly complex interdependencies between marital and fertility choices over the life course. Previous research has almost exclusively focused on marital status, although both fertility and marital histories can be expected to be closely intertwined with wealth accumulation processes across the life course.

Third, while most research on the role of family dynamics for economic wellbeing inspected household-level wealth due to data restrictions, we examine the personal wealth of household members as an under-researched dimension of economic wellbeing that may provide additional evidence of potentially gendered effects. We define personal net wealth as personally owned assets—solely owned or the personal share of joint assets—minus personal liabilities. We therefore acknowledge research that has questioned the unitary household model and the idea that all resources are fully shared and pooled among household members (e.g. Bennett, 2013; Joseph & Rowlingson, 2012). Although joint money management has been shown to be particularly likely within traditional stable marriages with children, previous quantitative and qualitative research has highlighted substantial within-couple wealth inequalities and particularly individualised money management approaches in more complex families, for example, following remarriage (Amuedo-Dorantes et al., 2011; Burgoyne & Morison, 1997; Grabka et al., 2015).

Empirically, we deploy longitudinal data from the West German sample of the German Socio-Economic Panel Study (SOEP, v34, waves 2002–2017). To establish typical family trajectory patterns, we use multichannel sequence analysis and cluster analysis of childbearing and marital histories spanning ages 16 through 50. To this end, we use retrospective life history information for men and women born between 1943 and 1967 who were aged 50–59 between 2002 and 2017. Using the identified set of family patterns, we predict disparities in personal wealth ranks at pre-retirement age (measured at ages 50–59) using OLS regression (*N* = 7004).

## Previous Research

Incipient previous research on disparities in *household-level* wealth by marital status unequivocally finds that, compared to ever experiencing a divorce, a continuous marriage is associated with higher wealth levels between ages 50 and 61 (Ulker, 2008; Wilmoth & Koso, 2002; Zissimopoulos et al., 2015).^2^ According to Zissimopoulos et al. (2015), marital dissolutions before the age of 26 were overall less detrimental for household wealth in older age—particularly for women—than dissolutions later in life. While being remarried at older ages was found to have partially restored household wealth compared to respondents who stayed divorced until old age, serial union dissolution severely penalised wealth in old age (Ulker, 2008; Wilmoth & Koso, 2002; Zissimopoulos et al., 2015).

By focusing solely on household-level wealth, previous studies may have underestimated gender inequalities within and between different family types as they assumed that all household resources are shared equally. Grabka et al. (2015) and Kapelle and Lersch (2020), however, illustrate substantial within-couple wealth inequalities that question the approach of previous research. Novel research by Lersch (2017) examines German panel data to scrutinise personal-level and household-level wealth differences across currently married, remarried, and divorced respondents between ages 50 and 75. Results show that while continuously married respondents have the highest personal and per capita wealth, men benefit more from continuous marriage than women with regard to their personal wealth levels at older ages. Although gender differences are statistically non-significant for remarried respondents, coefficients indicate that men may benefit slightly more from remarriage than women, compared to never married men and women. Across all wealth measures, Lersch (2017) finds that currently divorced respondents have the lowest levels of wealth in older age. Being divorced at older ages was thereby associated with marginally lower wealth for women than men.

The presence and number of dependent children is closely interlinked with parents’ marital status, but such intersections across family domains have only been partially addressed in wealth research. For the USA, Ulker (2008) finds that unmarried women’s, and married men’s and women’s per capita wealth at older age was negatively associated with the number of living children they had, while the number of children did not have a substantial effect on unmarried men’s per capita wealth. Despite addressing key intersections between fertility and marital status, the fact that these family statuses were measured in older age ignores the heterogeneous pathways that lead to the same marital status and final descent. Being unmarried at older ages may reflect a diverse range of marital histories from lifelong singlehood to highly disrupted marital patterns. Similarly, in the research by Ulker (2008), it was unclear whether married couples were in a first-time or higher order marriage or at what point in the life course family and fertility transitions took place.

The analysis of intersections between fertility and marital histories (which consist of all previous transitions between family statuses) including their timing and sequencing is critical to our understanding of the association between family life courses and wealth at older ages. This claim is supported by previous research that has illustrated that relevant variation in wealth exists across a range of marital states and fertility transitions during early and mid-adulthood (e.g. Lersch et al., 2017; Lusardi et al., 2001; Maroto, 2018). For instance, Lersch et al. (2017) show that a non-marital first birth or early birth compared to marital or late first birth are associated with substantially lower wealth growth rates for men and women although parenthood is overall more detrimental for women’s wealth accumulation than men’s. Whether these early wealth inequalities widen or narrow over time as children get older and form independent households or as other family transitions occur in the life courses of parents is still unclear.

## Theoretical Framework

In line with arguments about the origin and development of intra-cohort inequalities (Dannefer, 2003), disparities in wealth at older ages can be understood as an outcome of age differentiation: for a given birth cohort, the capacities and resources that contribute to the accumulation of wealth progressively differ among individuals as they age. According to the life course approach (Mayer, 2004), the rate of differentiation can be explained by (1) transitions, roles and experiences in multiple life domains (e.g. employment, family, etc.), (2) the linked experiences of others (e.g. contact with and support from family), and (3) the opportunities and constrains embedded in the socio-historical contexts of individuals’ lives.

Along these lines, the current paper explores how marital and parental roles enacted over the life course are associated with older-age wealth inequalities of West German men and women born between 1943 and 1967. In our study context, a nuclear family arrangement (i.e. husband and wife and their biological children) was demographically dominant at mid-adulthood, and was considered an economically-enhancing and socially-idealised family setting (Trappe et al., 2015).^3^ Nevertheless, the cohorts under study were the first to be exposed to rising de-standardisation trends in employment and family life courses. Despite the demise of marriage, marital childbearing, and marital stability among the cohorts under study, the absence of key structural elements of the standard family sequence, such as a traditional nuclear family arrangement throughout or over a large span of an individual’s life course, was deemed less beneficial or even a hindrance to the achievement of subjective and objective wellbeing including financial prosperity. Ours is one of the first studies to address whether marital and parental roles and transitions from early adulthood until pre-retirement age associate with economic wellbeing, empirically assessing wealth levels in older age as a potential outcome. Although it is not our aim to test the concrete mechanisms, we acknowledge that several complementary explanations would support the potential associations between family trajectories and wealth levels of men and women in older ages, which we further elaborate on in the following.

### Wealth Benefits and Penalties Associated with Family States and Transitions

As part of the standardisation of life courses in the post-war period, a relatively early marriage was incentivised leading to a spike in marriage rates. Although marriage rates have since declined with cohabitation and divorce common, a stable first marriage is still associated with a range of wealth-enhancing mechanisms for both men and women (Lersch, 2017). This marriage wealth premium is shaped, firstly, by greater economies of scale and institutional benefits (i.e. tax, pension, or insurance benefits) that enable higher saving rates. Secondly, social norms around marriage explicitly emphasise saving for a joint future, highlighting long-term commitment, and increasing intergenerational transfers. High levels of commitment and perceived longevity of the marital institution additionally provide an environment in which sharing, and resource integration are perceived as low risk. This increases the likelihood of investing in assets that may provide higher returns in the long-term and over time compounded interest effects may exponentially increase wealth as a form of cumulative advantage. Thus, the duration of marriage matters with marriage at a normative age (i.e. after completion of education and not too late in the life course) associated with an optimal time to maximize wealth benefits associated with marriage.

Although both men and women economically benefit from marriage, it needs to be considered that women commonly have a lower wealth accumulation potential compared to men, *inter alia*, due to a pervasive gender pay gap and women’s lower access to wealth-relevant fringe benefits (Chang, 2010). While women’s lower potential can to some degree be compensated by their male partners, not all resources are shared and pooled even within marriage (Bennett, 2013; Joseph & Rowlingson, 2012). Additionally, men are often older at partnership formation which may be associated with more individual wealth at partnership formation for men.

Departure from a stable marriage either through marital dissolution or refraining from marriage would result in a partial or full loss or lack of marital premiums. It is also worth noting that marital dissolution is often associated with substantial immediate wealth losses due to the costs of separation and divorce, including expenses for the administrative process itself, possible relocation, and the division of marital wealth (Kapelle & Baxter, 2021). The earlier a marital dissolution takes place, the lower are potentially wealth penalties as couples had less time to jointly accumulate wealth which would result in lower administrative divorce costs or a more straightforward division of assets. While marital premiums may be restored during a remarriage, such premiums are expected to be lower due to the greater financial independence of individuals in higher order marriages and potential financial commitments to ex-spouses (Burgoyne & Morison, 1997).

In addition, marriage entries and exits are socially stratified and vary across wealth levels and relevant characteristics including labour market income, employment status, education, or families’ socio-economic origins (Eads & Tach, 2016; Gibson-Davis et al., 2005; Schneider, 2011). Such economic prerequisites for family transitions have been more salient for men than women (Xie et al., 2003). On average, those who married tend to have wider access to social and economic resources than those who did not, and thus, financial prosperity would have arguably been higher for the married even in the absence of marriage.

Parenthood is associated with a range of direct and indirect costs. Despite some institutional economic support for parents (e.g. child allowance or tax benefits for parents), the responsibility to cover child-related costs largely rests on parents, which can limit their potential to accumulate wealth. Direct costs relate to expenses for daily living (e.g. food, rent), and fees for child care and education (Bradbury, 2011). Indirect financial costs of childrearing particularly emerge for women due to related career breaks (Budig & England, 2001), which restrict women’s current and future income and thus wealth accumulation potential (Lersch et al., 2017). The latter follows from a culturally-persistent and institutionally-supported male breadwinner model, where men are meant to provide economic resources for the household while women are the main caregivers. However, ideas of full financial pooling and sharing have been shown to be flawed (e.g. Bennett, 2013), and wealth advantages of marriage entry are likely reduced through child-related career disruptions.

For an average family size, direct childbearing costs can be offset, to a large extent, in the context of a stable parental marriage. First, married parents often fulfil some economic prerequisites for childbearing, particularly fathers. To provide financial security for mother and child while also ensuring an ideal setting for child socialisation, it was commonly thought that childbirth ought to take place within marriage and preferably only after men achieved a consolidated position in the labour market (Oppenheimer, 1988). This is in line with middle-class ideals from the 1950s that supported a standardised family life course. Second, actual or anticipated childbearing generates long-term savings incentives to cover child-related costs, which continue even after children are no longer dependent on parents (Lusardi et al., 2001). Third, married parents often benefit from intergenerational financial transfers as a form of social support (Leopold & Schneider, 2011), which can additionally increase wealth levels.^4^

In our study context, marriage was the normative family environment for childbearing^5^ (Le Goff, 2002), and desire for children influenced marital transitions and their timing (Baizán et al., 2004). In contrast, due to the social stigma of out-of-wedlock parenthood, long-term cohabitation of parents was uncommon and often ended either in marriage or single parenthood (Le Goff, 2002). The likelihood of either pathway is socially stratified, with economically more advantaged and established parents transitioning to marriage, and younger parents with an incomplete education and lower income separating, which highlights the importance of the timing of parenthood (Upchurch et al., 2002). Among married parents, divorce is also more likely among financially stressed individuals highlighting selection out of marriage (Eads & Tach, 2016). Divorce itself is likely associated with a range of wealth-depleting expenses (Kapelle & Baxter, 2021). Overall, single parents—either due to divorce or to out-of-wedlock births—lack or lose the economic advantages of marriage, including financial transfers between parents (Eickmeyer et al., 2019) and across generations (Manning et al., 2003). As children commonly reside with mothers, single parenthood often restricts women’s economic potential as they bear a larger share of the direct child costs, and they incur indirect costs of employment restrictions related to taking care of children. Child alimony paid by the non-residential fathers is relatively low and does not affect poverty risks for fathers (Hakovirta et al., 2019). Nevertheless, regular child alimony payments may have the potential to reduce surplus income and thus savings for men.

### Study Objectives

Consistent with the *cumulative advantage/disadvantage theory* (O’Rand, 1996), we extend the above-mentioned arguments about wealth-advantageous and wealth-penalising family states and transitions to explain differential wealth outcomes in older age. We argue that wealth disparities between individuals at older ages can be a function of individuals’ wealth advantages and penalties, accumulated through their family behaviour at younger ages. Additionally, wealth disparities can be the result of socio-economically stratified family behaviour, where individuals with higher potential to accumulate wealth are disproportionately more likely to transition into (and less likely to transition out of) specific family roles. In particular, deviations from the culturally and institutionally-supported *standard* trajectory of continuous marriage combined with moderate fertility may lead to lower rates of wealth accumulation and to increasing wealth disparities because wealth-enhancing mechanisms are either disrupted or absent. Regarding our empirical analysis, we expect that *having enacted a standard family trajectory is associated with greater wealth at ages 50–59, while life courses deviating from the standard family trajectory can be expected to be linked to less wealth-enhancing structures and thus lower wealth at these ages*.

Non-standard family trajectories are, however, diverse, and heterogeneous with regard to the type of deviation from the standard trajectory. Some trajectories might only deviate slightly, regarding the occurrence, timing or sequencing of family transitions that conform to the standard. This may be, for example, due to the postponement of marriage or the decision to have one child less than the average. One can expect small to trivial wealth disparities when trajectories depart only moderately from the standard, because most wealth enhancement mechanisms will still be in place and only small, if any, wealth penalties will be incurred. Some other trajectories might feature substantial deviations, ranging from the complete absence of family transitions to a highly complex set of transitions that often include non-typical, disadvantaged family arrangements such as single parenthood and patchwork families. One can expect larger wealth disparities when trajectories deviate substantially from the standard, because wealth-enhancing mechanisms associated with the enactment of the standard trajectory are absent or disrupted and additional wealth penalties will be incurred and may accumulate, depending on the complexity of family trajectories (e.g. repeated divorce, childbearing with multiple partners). We thus expect that *wealth levels will vary substantially between groups of non-standard family trajectories with larger deviations from the standard pathway associated with higher wealth penalties and smaller deviations associated with substantially lower wealth penalties*.

Finally, wealth accumulation potentials likely differ for men and women over their life courses. Gender wage inequalities and access to employment-related wealth building tools are cited as the main drivers of these disparities (Sierminska et al., 2010). While penalties partially emerge based on occupational segregation and an undervaluing of female-dominated industries (Hakim, 1992; Perales, 2013), family roles enacted over the life course also matter. Women’s wealth accumulation potential is substantially inhibited by parenthood-related career breaks (Lersch et al., 2017). The degree to which these potential disadvantages develop into lasting penalties likely differs according to the availability and consistency of their partner’s (financial) support.

## Method

### Data

We use longitudinal survey data from the German Socio-Economic Panel study (SOEP; https://doi.org/10.5684/soep.v34). The SOEP is a nationally representative household panel study that has been administered yearly since 1984 in West Germany and has since been extended several times (Goebel et al., 2019). The data are particularly suitable for our research purposes as they (i) contain retrospective information on detailed marital and childbearing histories from late teen ages to date, and (ii) collect comprehensive *personal*-level wealth data in four survey waves (2002, 2007, 2012, and 2017).^6^ In our analyses, we rely on wealth data that were edited and imputed by the SOEP survey team (Grabka & Westermeier, 2015). Building on the imputed wealth data, we additionally address item nonresponse in relevant analytical variables—except for marital and fertility history data^7^—through multiple imputation using Stata’s *mi* procedure (version 16). Estimation results from five imputed data sets are combined using Rubin’s rule (Rubin, 1987).

### Sample

**Fig. 1:**
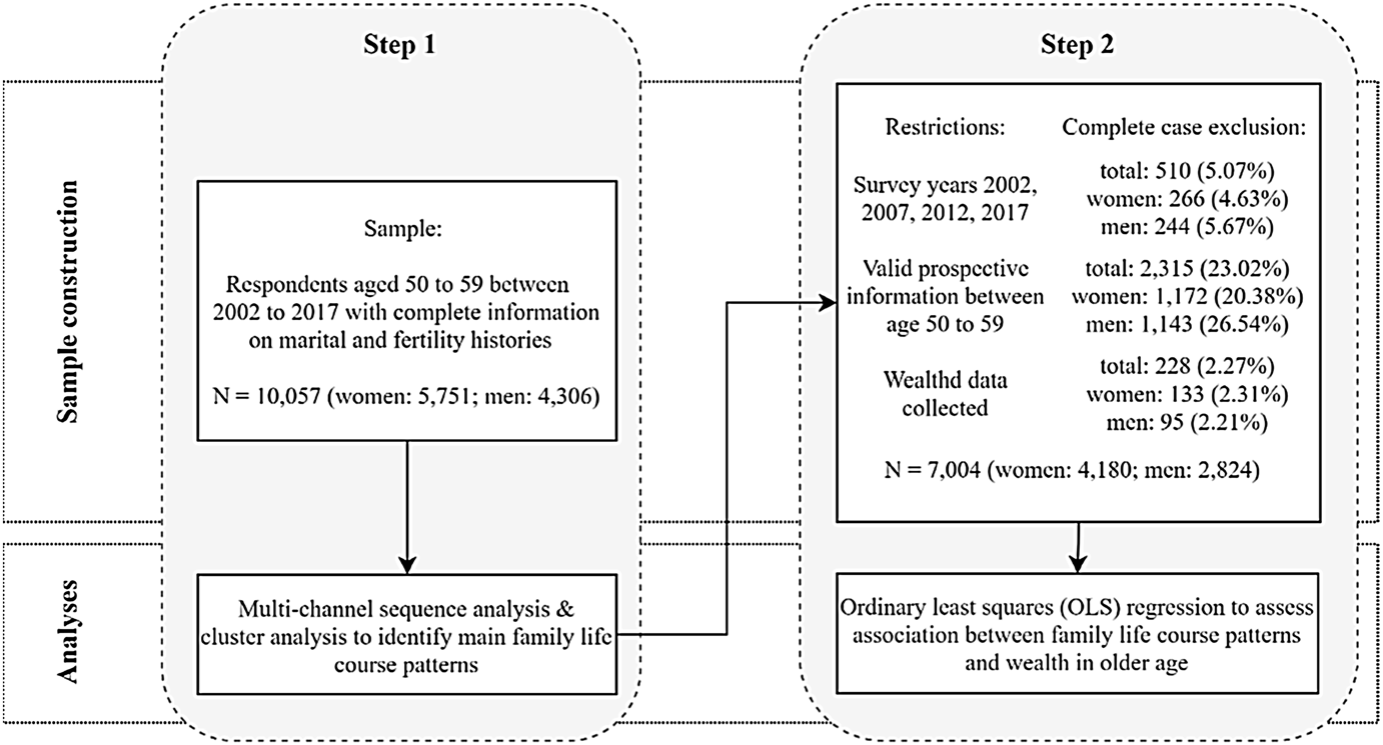
Sample selection process and analytical strategy diagram

Our sample construction follows a two-step process resulting in an initial sample that is used to establish a typology of family patterns and a more restricted sample for the outcome regression (see Fig. 1). This two-step process is necessary to ensure that our family typology, which is based on the initial sample, reflects the underlying family life course patterns for the *entire* cohort of interest (i.e. respondents born between 1943 and 1967), while our regression sample can only consider respondents with valid wealth information. In the following paragraphs, we discuss the sample construction in more detail.

First, we select respondents who were aged 50–59 at any time between 2002 and 2017 and who provided complete retrospective marital and fertility histories from ages 16 to 50. We decided to focus on respondents in their 50 s as wealth penalties and advantages accumulate over the life course and become particularly visible in older age (Hurd, 2002). Further, wealth levels can be expected to peak at this time in preparation for retirement in line with the life cycle hypothesis (Alessie et al., 1997; Milligan, 2005; Modigliani, 1988). Additionally, the focus on pre-retirement age for a large proportion of respondents was desirable because wealth decumulation during retirement as well as retirement entry itself are highly socially stratified (Alessie et al., 1997; Montalto et al., 2000). Thus, if we find substantial wealth inequalities in association with family life course patterns, this would highlight that respondents are differently prepared for retirement or even likely to enter retirement. Although the legal retirement age for the cohort of interest is 65–67, actual retirement entry often occurs earlier (Deutsche Rentenversicherung Bund, 2018). Finally, our focus on respondents up to the age of 59 reduces the influence of older age family transitions, specifically widowhood. Based on these criteria, our sample contains 10,057 respondents with 5751 women and 4306 men. As men’s retrospective fertility data have only been collected for men who entered the SOEP in 2000 or later, our sample includes fewer men than women.^8^ Overall, this sample is used to generate our typology of family life courses.

As previously mentioned, further restrictions of our initial sample are necessary for the multivariate analysis. First, we restrict the sample to survey years 2002, 2007, 2012, and 2017 in which wealth data are collected. This leads to an exclusion of 510 respondents (266 women, 244 men). Next, we exclude 2315 respondents (1172 women, 1143 men) without valid prospective information between age 50 and 59. Finally, respondents from whom no wealth information was collected are excluded reducing the sample by 228 respondents (133 women, 95 men).^9^ After these exclusions, our final regression sample consists of 7004 respondents of which 4180 are women and 2824 are men.

### Measurements

#### Wealth Measures

Our outcome measure, *total personal net wealth,* is defined as the sum of all personally owned assets minus liabilities. Asset components in the SOEP include property assets, tangible and financial assets, private pensions, business assets and collectables, while liabilities refer to consumer credits or mortgage debts. For each household member aged 17 and older, SOEP personal wealth data have been collected in a three-step process: (1) a filter question is used to assess ownership of a certain wealth component; (2) the total market value of held wealth components is recorded; and (3) for jointly held wealth components, respondents are asked to provide the share they co-owned. Our outcome measure thus explicitly includes the personal share of any assets and liabilities that were owned with other individuals. Personal net wealth is adjusted for inflation using the consumer price index set to 2015 prices. As wealth data are highly right-skewed, we transform total personal net wealth by ranking individuals by their personal net wealth separately for each wealth survey year but jointly by gender. The final rank measure provides a straightforward indication of individuals’ positions within the wealth distribution. Ranging from 0 to 100, the rank measure indicates the proportion of respondents that have less wealth than the individual considered.^10^

#### Family Trajectory Patterns

Our main explanatory variable is a categorical measurement of major family life course trajectories. We define the family trajectory as a sequence or succession of family states over time and build a typology deploying sequence analysis (see analytical strategy, below).

To compile respondents’ family sequences, we use biographical information on respondents’ marital status and childbearing status between ages 16 and 50. This information was collected prospectively and retrospectively for life periods pre-dating panel entry. We build one sequence of yearly marital states and one sequence of yearly childbearing states per respondent. The marital sequence captures four relevant partnership situations: “Single, never married”, “Married”, “Previously married”, and “Remarried”. The “Single, never married” state includes episodes of pre-marital singlehood as well as of pre-marital cohabitation. The “Married” state refers to the first marital episode. “Remarried” refers to higher order marital episodes, though most of them are second order. “Previously married” consists mostly of separated—from a marriage—or divorced individuals,^11^ who might be living in a single-headed household or cohabiting with a partner. Despite the increasing focus on non-marital cohabitation in recent studies, this information is not available retrospectively in the SOEP. Additionally, long-term cohabitation only gained acceptance in more recent cohorts than those included in the study and was commonly not recorded in West Germany due to its negligible role in the life courses of the cohorts of interest (Le Goff, 2002).

The childbearing sequence consists of five categories capturing the number of children: “Childless”, “1 child”, “2 children”, “3 children”, and “4+ children”. Each category indicates the reported number of the respondents’ ever born or adopted children at a given age. Since no information on household composition is available in the biographical questionnaire, states in the childbearing sequence do not consider whether or for how long children lived in the household. Despite this, the childbearing sequence is illustrative of whether individuals followed a normative sequence regarding the quantum and tempo of childbearing.

### Analytical Strategy

Our analytical approach progresses in two stages. First, we use methods for the analysis of sequence data to identify major family life course patterns. Next, we deploy regression analyses to assess the association between the heterogeneity in the identified family patterns and personal wealth ranks in later life.

To establish a typology of family trajectories, we use multichannel sequence analysis (MCSA) (Gauthier et al., 2010) in the *TraMineR* package (Gabadinho et al., 2008) of the software R (version 3.3.3) using the above-mentioned state sequences for the marital and childbearing domains as the units of analysis.^12^ To account for cross-domain interdependence, MCSA averages domain-specific transformation costs of Optimal Matching (OM) distances to subsequently compute dissimilarities across pairs of sequences. We use a novel cost structure for OM distances proposed by Studer and Ritschard (2016), where substitution costs are derived from the data as the Chi-squared distance between conditional state distributions in future periods.^13^ This cost structure considers that different family states at a given age are more alike if followed by shared family states at later ages, and thus, the resulting pairwise dissimilarities are more sensitive to changes in structural aspects of family life courses predicted by de-standardisation theses than typical OM distances.^14^

Building on the distance matrix resulting from the MCSA, we identify the specific family patterns that are relevant in the study population to address the significance of the standard trajectory, and to identify consistent patterns that deviate from each other and assess the specific aspects of deviance. To this end, we employ cluster analysis on the matrix of pairwise distances to group sequences to generate a typology of family patterns. We use a Ward link to generate internally homogeneous groups that are simultaneously heterogeneous between themselves. The decision on the number of groups is based on empirical fit measures using cluster stopping rules (see Figure S.3. in the supplementary material for a visualisation of cluster cut-off criteria).

Prior to our regression analyses, we assess key differences across major family patterns regarding family transitions and socio-economic compositions within a descriptive analysis. We additionally provide untransformed mean personal wealth levels across family patterns as a first indication of our association of interest. We then formally predict the association between specific family patterns and wealth ranks using OLS regressions with cluster-robust standard errors. Regressions include an interaction between gender and family patterns to account for different outcomes between men and women. As previously mentioned, we use imputed data, and thus estimation results from five imputed data sets are combined using Rubin’s rule (Rubin, 1987). All estimates are adjusted a range of baseline confounders that partially predict both selection into certain family pathways and base-level wealth. A detailed description of these confounders is provided in the supplementary material (see section S.2.3). Regression analysis was performed using the statistical software Stata (version 16).

## Results

### Heterogeneity in Family Trajectories

**Fig. 2:**
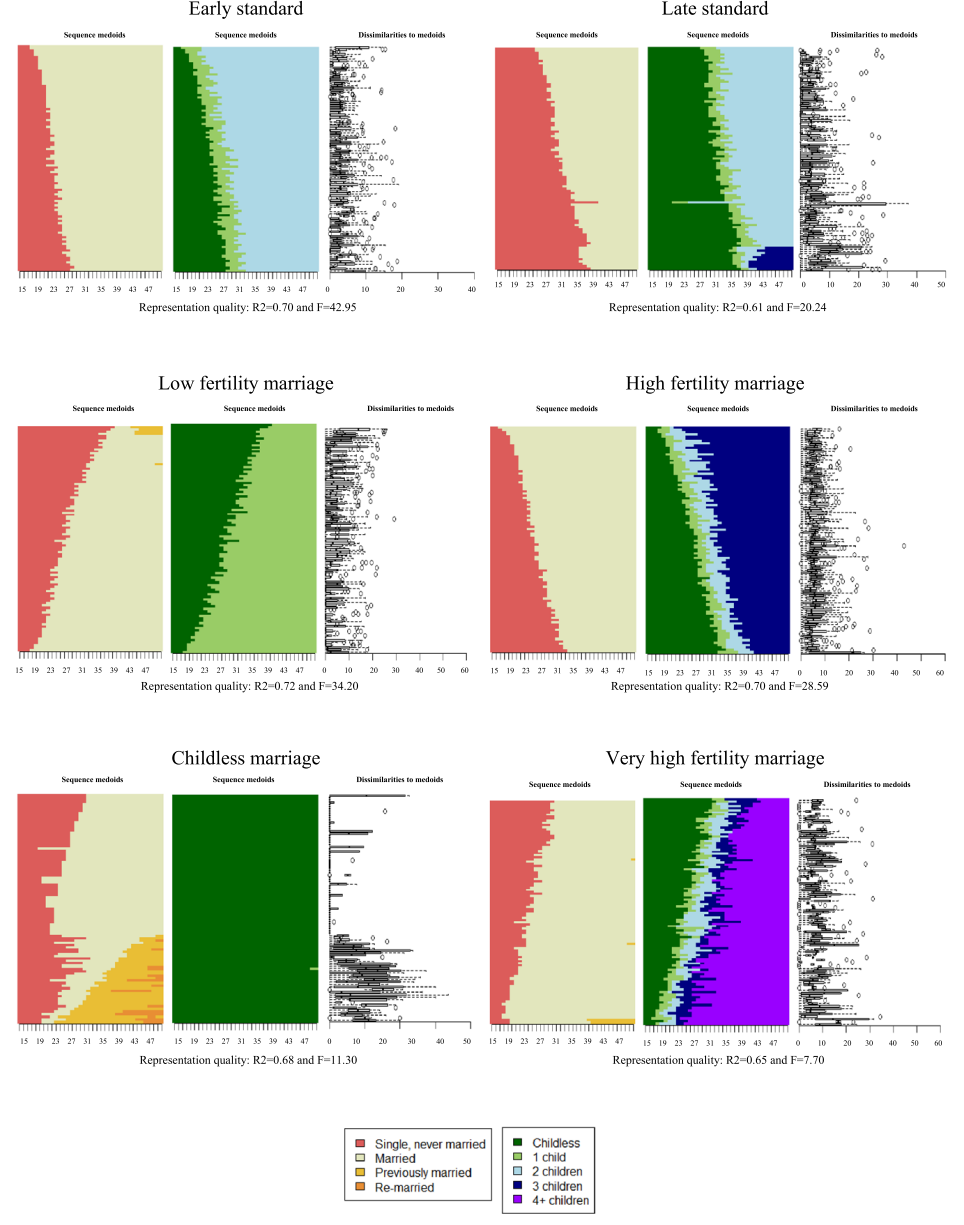

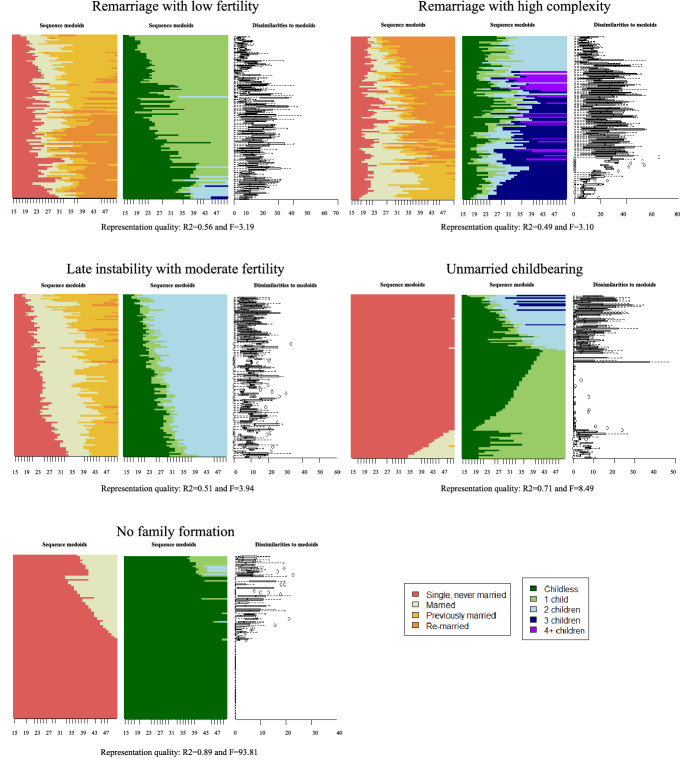
Relative frequency sequence plots of the identified major family patters. *Notes:* Retrospective data on marital and fertility histories are from the Socio-Economic Panel Survey v34 (2002–2017; non-imputed)

We describe the heterogeneity in family trajectories of study cohorts by clustering individual sequences in major family life course pathways. Multiple cluster cut-off criteria supported either 3 or between 9 and 11 clusters (see Figure S.3. in the supplementary material). We chose the 11-cluster solution as a trade-off between internal consistency of clusters and substantive interdependence in marital and childbearing life courses for the German cohorts under study. An assessment of cluster quality indicates that the level of within-cluster homogeneity is moderate (see section S.2.5 of the supplementary material for further detail on the cluster quality). Figure 2 provides a visual illustration of the 11 pathways.^15^ Pathways were ordered based on expected divergence from the standard family life course, starting with patterns that feature stable marriage and descending to patterns that feature marital instability or lack of marriage. We additionally sorted by the similarity of fertility behaviour to the standard trajectory. To provide a thorough understanding of these eleven major pathways, along with the description of the sequence structure of family events, we assess their average socio-demographic and occupational compositions (see Table 1). While the following description and naming of the 11 clusters focuses on the most prominent features for each identified cluster, it should be noted that clusters may include relevant within-cluster heterogeneity as also visible in Fig. 1 and highlighted in Table 1.

**Table 1:** Summary indicators of major family patterns

	Family patterns											Total
	Stable marriage						Marital instability			No marriage		
	Early standard	Late standard	Low fertility marriage	High fertility marriage	Childless marriage	Very high fertility marriage	Remarriage with low fertility	Remarriage w/ high complex	Late instability w/ mode rate fertility	Unmarried childbearing	No family formation	
Relevant within-cluster heterogeneity/ Sub-population within cluster characterized by	Heterogeneous age at marriage and childbearing	Up to 3 children	Heterogeneous age at marriage and childbearing	Heterogeneous age at marriage and childbearing	Marital disruption, remarriage, heterogeneous age at marriage	Heterogeneous age at marriage and childbearing	Childbearing in remarriage, never remarried	Higher order dissolution, multi-partner fertility, never remarried	Short first union, re-marriage	Heterogeneous age at childbearing, late marriage	Late marriage and childbearing	
	mean/(SE)	mean/(SE)	mean/(SE)	mean/(SE)	mean/(SE)	mean/(SE)	mean/(SE)	mean/(SE)	mean/(SE)	mean/(SE)	mean/(SE)	mean/(SE)
*Wealth levels*												
Personal net wealth	179.88	223.16	182.92	202.03	185.59	140.95	116.56	114.46	146.34	103.66	175.39	174.50
	(280.72)	(387.14)	(307.44)	(385.32)	(304.07)	(329.66)	(203.94)	(356.32)	(315.84)	(242.18)	(355.44)	(331.20)
*Basic demographics*												
Female	0.72	0.41	0.65	0.61	0.63	0.66	0.65	0.71	0.73	0.71	0.42	0.61
Migration background	0.14	0.10	0.09	0.12	0.08	0.21	0.10	0.10	0.10	0.10	0.07	0.11
Cohort												
1943–1950	0.34	0.16	0.29	0.20	0.25	0.22	0.28	0.19	0.20	0.07	0.14	0.23
1951–1958	0.41	0.29	0.39	0.35	0.41	0.39	0.39	0.39	0.33	0.21	0.36	0.36
1959–1966	0.24	0.54	0.32	0.44	0.33	0.39	0.33	0.42	0.48	0.72	0.49	0.41
Number of siblings	2.16	2.00	1.86	2.31	1.72	3.03	1.97	2.40	1.94	2.18	1.87	2.09
	(1.86)	(1.72)	(1.61)	(1.87)	(1.65)	(2.30)	(1.92)	(1.98)	(1.69)	(1.74)	(1.63)	(1.81)
Parental education												
Low	0.22	0.12	0.17	0.21	0.14	0.26	0.20	0.24	0.18	0.14	0.12	0.18
Middle	0.73	0.70	0.76	0.68	0.76	0.62	0.75	0.68	0.72	0.74	0.75	0.72
High	0.05	0.19	0.07	0.12	0.10	0.13	0.05	0.08	0.10	0.12	0.14	0.10
Parental occupational prestige	39.15	44.78	40.83	41.80	42.28	41.55	39.30	39.43	41.27	42.00	43.65	41.59
	(10.99)	(12.80)	(11.55)	(12.83)	(12.99)	(12.45)	(11.36)	(11.44)	(11.92)	(13.35)	(13.09)	(12.34)
*Family pattern up to age 50*												
Age at first birth	24.51	32.64	29.47	26.51	44.64	24.67	28.39	23.25	25.62	30.74	41.27	27.99
	(3.29)	(3.64)	(5.63)	(4.58)	(3.37)	(4.49)	(7.35)	(4.57)	(4.25)	(6.65)	(3.40)	(6.05)
Number of children	2.00	2.19	1.02	3.00	0.04	4.56	1.22	3.14	2.06	1.47	0.38	1.84
	(0.03)	(0.56)	(0.15)	(0.06)	(0.29)	(0.98)	(0.53)	(0.99)	(0.29)	(0.81)	(0.82)	(1.22)
Unmarried childbearing	0.20	0.28	0.17	0.27	0.01	0.37	0.42	0.61	0.40	1.00	0.09	0.27
Multi-partner childbearing	0.00	0.01	0.01	0.01	0.00	0.03	0.07	0.52	0.13	0.00	0.00	0.04
Age at first marriage	23.47	31.96	27.01	25.89	26.44	24.86	23.82	22.11	24.91	43.89	40.69	27.34
	(2.63)	(4.07)	(4.98)	(4.24)	(3.94)	(3.89)	(3.65)	(3.00)	(3.87)	(4.77)	(4.40)	(6.31)
Ever married	1.00	1.00	1.00	1.00	1.00	1.00	1.00	1.00	1.00	0.24	0.53	0.92
Ever divorced	0.04	0.08	0.22	0.09	0.38	0.11	0.96	0.94	0.86	0.02	0.03	0.21
Ever remarried	0.00	0.01	0.05	0.01	0.17	0.02	0.73	0.83	0.33	0.00	0.00	0.10
*Human capital–men*												
Education												
Low	0.04	0.03	0.04	0.06	0.05	0.09	0.02	0.11	0.03	0.07	0.07	0.05
Middle	0.62	0.39	0.55	0.37	0.51	0.43	0.66	0.61	0.59	0.59	0.49	0.49
High	0.34	0.58	0.41	0.57	0.44	0.48	0.33	0.28	0.38	0.35	0.45	0.46
Full-time employment years	31.99	28.25	30.52	29.37	30.57	28.89	30.32	28.52	30.10	28.50	26.84	29.24
	(5.61)	(6.44)	(6.45)	(6.35)	(6.33)	(6.90)	(7.21)	(7.93)	(6.38)	(7.66)	(8.08)	(6.95)
Non-/Un-employment episodes	0.30	0.47	0.53	0.44	0.51	0.58	0.69	0.95	0.64	0.92	0.77	0.55
	(0.66)	(0.87)	(0.91)	(0.84)	(0.88)	(1.02)	(1.17)	(1.22)	(0.98)	(1.25)	(1.11)	(0.96)
Occupational prestige (mode)	46.53	50.68	48.10	50.08	48.59	48.10	47.24	42.62	45.72	43.79	47.77	48.25
*Human capital–women*												
Education												
Low	0.14	0.04	0.06	0.15	0.08	0.25	0.11	0.25	0.13	0.10	0.07	0.12
Middle	0.68	0.47	0.70	0.52	0.60	0.44	0.69	0.61	0.66	0.57	0.46	0.60
High	0.18	0.49	0.23	0.33	0.32	0.31	0.19	0.14	0.22	0.32	0.47	0.28
Full-time employment years	11.35	12.51	16.24	9.61	24.69	7.09	18.95	12.66	15.37	16.52	24.39	14.48
	(9.82)	(8.02)	(10.62)	(8.46)	(10.35)	(7.99)	(10.23)	(9.91)	(9.12)	(9.88)	(9.97)	(10.78)
Non-/Un-employment episodes	2.01	2.10	1.70	2.18	1.27	2.17	2.32	2.56	2.42	2.19	1.30	1.97
	(1.34)	(1.34)	(1.32)	(1.35)	(1.35)	(1.40)	(1.43)	(1.24)	(1.36)	(1.38)	(1.41)	(1.39)
Occupational prestige (mode)	40.71	48.46	44.26	42.65	47.15	41.22	43.62	39.48	41.71	44.51	48.97	43.61
*Observations*	1791	1307	1325	1237	571	468	342	430	452	375	1105	9403
*Individuals*	1285	1006	976	934	419	347	251	309	334	305	838	7004
*% respondents*	18.35	14.36	13.93	13.34	5.98	4.95	3.58	4.41	4.77	4.35	11.96	100.00

Data are from Socio-Economic Panel Survey v34 (2002, 2007, 2012, 2017; imputed, unweighted. See Table S.3 for descriptive result using non-imputed data

We identify two patterns that reflect the anticipated standard: the *Early Standard* pattern (18.4 percent of the sample) and the *Late Standard* pattern (14.4 percent of the sample). While both patterns consist of long, uninterrupted marriage trajectories with two children, they differ in the timing of transitions into marriage and parenthood. Whereas respondents in the *Early Standard* pattern enter marriage on average at age 23 and parenthood shortly after at age 25, respondents in the *Late Standard* pattern experience these family transitions on average at age 32 and 33, respectively. The two patterns differ by gender, cohort and to some degree by human capital characteristics. In line with common age differences between men and women at family formation and an increasing postponement of family formation in later cohorts, the *Early Standard* pattern is more common for women (21.1 percent of all female sample respondents) and respondents from the earlier study cohorts. The *Late Standard* pattern is more common among men (21.0 percent of all male sample respondents) and respondents from the latest considered cohort. The *Late Standard* pattern also contains respondents with the overall highest educational achievements and occupational prestige. It is worth noting that in both patterns men show substantially higher human capital and occupational achievements than women.

Four other family patterns are largely characterized by stable marriage, but they deviate from the standard patterns particularly regarding fertility levels and range between the two standard patterns in terms of age at family transitions. In combination, these patterns garner 38 percent of the respondents’ sample. Two patterns diverge only slightly from the standard patterns’ fertility behaviour: *Low fertility marriage* (13.9%) and *High fertility marriage* (13.3%). It is worth noting that marriage entry and first birth take place earlier in the latter pattern (with three children), compared to the former pattern (with one child). While the human capital achievement for men in the two groups are comparable, the three-child pattern features lower human capital attainments for women than the one-child pattern. Last, two patterns present fertility behaviour that contrasts with the standard patterns: the *Childless marriage* (6.0%) and the *Very high fertility marriage* (5.0%). Beyond no fertility, respondents within the *Childless marriage* pathway are also characterized by high levels of human capital for both men and women. The very high fertility pattern consists of trajectories with four or more children, and children often born out-of-wedlock. This pattern is common among respondents with a migration background and those from larger families themselves. It is also associated with the lowest human capital for women. Overall, all continuously married patterns—except the patterns with three or more children—display above-average personal wealth levels.

The next three patterns (12.8%) feature marital instability and therefore discontinuity of marital premiums over the life course: *Remarriage with low fertility* (3.6%), *Remarriage with high* c*omplexity* (4.4%) and *Late instability with moderate fertility* (4.8%). Early marriage and out-of-wedlock childbearing are common in all these patterns. While the first two patterns feature a high proportion of remarriage and relatively short first marriages, the last pattern features lower levels of remarriage and higher duration of the first marriage. The three patterns also differ regarding their fertility patterns. Respondents in the *Remarriage with low fertility* pattern and the *Late instability with moderate fertility* pattern have on average one or two children, respectively. The *Remarriage with high* c*omplexity* pattern features comparatively high fertility levels with on average three children, as well as high levels of multi-partner fertility. Men in the three clusters exhibit slightly below-average levels of human capital. Women’s attachment to full-time employment is above average in the two patterns with less complexity. Nevertheless, trajectories of marital instability with or without remarriage are characterized by substantially below-average levels of wealth.

The last two patterns (16.3%) deviate from the standard pattern as they largely lack marriage entry. Additionally, the two patterns differ in terms of fertility behaviour. The *Unmarried childbearing* (4.5%) pattern features childbearing at above-average age (first childbirth on average at 30.7 years). It is more common among women and is associated with average levels of human capital for women, but below-average levels for men. Respondents in this pattern hold the lowest levels of wealth overall. The pattern of *No family formation* (12.0%) features trivial fertility and marital levels. It is more common among men, for whom it is associated with below-average human capital. Women in this cluster show comparatively high levels of human capital. Overall, this pattern shows average wealth levels.

### Wealth Across Major Family Patterns

**Fig. 3:**
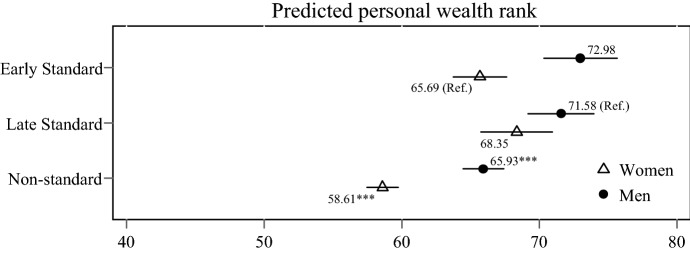
Predicted personal wealth rank of men and women aged 50–59 in the standard family pattern and the non-standard family pattern based on multivariable OLS regression models. *Notes:* Whiskers indicate 95% confidence intervals. Data are from the Socio-Economic Panel Survey v34 (2002, 2007, 2012, 2017; unweighted; multiply imputed). Models include control variables for age, migration background, birth cohort, number of siblings, parental education, parental occupational prestige, marital events after the age of 50 (marriage, divorce, widowhood). Full model results in Table S.4 in the supplementary material. *p < 0.05, **p < 0.01, ***p < 0.001 indicate whether coefficient is significantly different to reference (*Early standard* for women, *Late standard* for men) in regression

We move on to multivariate OLS regressions, which allow us to obtain better estimates of the study associations by adjusting for confounders while also clustering standard errors at the household level. We use the *Early standard* pattern as the reference pattern for women and the *Late standard* pattern as the reference pattern for men in our description of regressions results because those are the most prevalent patterns for men and women in line with ideas of women’s earlier family transitions compared to men. Differences in estimated wealth levels between the two patterns are marginal for men and women and thus results hold even if changing the reference category. As the first step, we examine differences in men’s and women’s wealth ranks between the *Standard* patterns and *Non-standard* patterns (i.e. a combination of all patterns other than the two standard patterns). Figure 3 shows predicted personal wealth ranks for men and women in each pattern, which also provides a straightforward illustration of gender differences in wealth levels. Results show substantially and statistically significantly lower personal wealth ranks for respondents who followed *Non-standard* patterns. As expected, women hold lower average wealth ranks than men with substantial gender gaps in the *Early standard pattern* and the *Non-standard* patterns. Less substantial gender differences are only evident between men and women in the *Late standard pattern*.

**Fig. 4:**
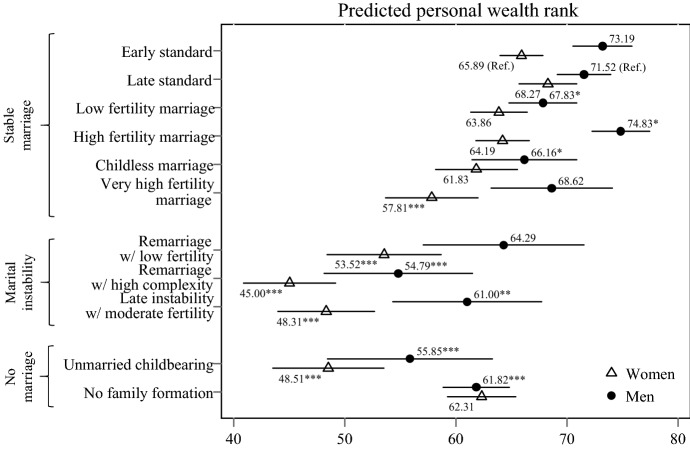
Predicted personal wealth rank of men and women aged 50–59 across the diversity of family patterns based on multivariable OLS regression models. *Notes:* Whiskers indicate 95% confidence intervals. Data are from the Socio-Economic Panel Survey v34 (2002, 2007, 2012, 2017; unweighted; multiply imputed). Models include control variables for age, migration background, birth cohort, number of siblings, parental education, parental occupational prestige, marital events after the age of 50 (marriage, divorce, widowhood). Full model results in Table S.4 in the supplementary material. *p < 0.05, **p < 0.01, ***p < 0.001 indicate whether coefficient is significantly different to reference (*Early standard* for women, *Late standard* for men) in regression

Figure 4 shows predicted wealth ranks across the two *Standard* patterns and specific *Non-standard* patterns for men and women. In addition to the two *Standard* patterns, we identified four family patterns that also feature a continuous marriage but depart from the *Standard* patterns particularly on fertility levels. These patterns are displayed at the top of the graph, below predictions for men and women in the *Standard* patterns. In line with our thesis of lower penalties for smaller deviations from a standard life course, for women, we find that the majority of patterns featuring smaller deviations are associated with a similar rank in the wealth distribution compared to the *Standard* patterns. Only the pattern with very high fertility levels is associated with substantially and significantly less wealth; 8 ranks lower compared to women in the *Early standard* pattern. For men, deviation from the standard pattern is associated with substantially lower penalties compared to women although even small differences are statistically significant for men. With 5 rank points lower average personal wealth, the *Childless marriage* pattern is associated with the highest wealth penalty for stably married men compared to men in the *Late standard* pattern.

Next, we move to the three family patterns that feature marital instability and thus higher deviation from the *Early* or *Late standard* pattern. We find that all marital instability patterns are associated with substantially lower personal wealth ranks for women; 12–21 lower ranks than the *Early standard* pattern. For men, we find similar results to those of women; ranks 7–17 points below the *Late standard* pattern for personal wealth. The lowest penalties in the marital instability patterns are experienced by men and women in the *Remarriage with low fertility* pattern while the highest penalties are experiences by men and women in the *Remarriage with high complexity* pattern. Women in the *Remarriage with high complexity* even rank the lowest across all patterns. It needs to be considered that substantial within-pattern gender differences to the disadvantage of women are visible in the three marital instability patterns.

The last two patterns feature unmarried family trajectories. In the personal wealth distribution, both men and women within the *Unmarried childbearing* pattern rank lower than men and women in any of the stable marriage patterns. Compared to the subsequent *Standard* pattern, men and women with family life courses that featured unmarried childbearing rank 16 and 17 points lower than men and women that followed a standard pattern. However, ranks for respondents in the *Unmarried childbearing* pattern are not statistically different to most of the marital instability patterns. The pattern of *No family formation* is associated with lower ranks than the *Standard* pattern for men, but not women. For men, wealth penalties associated with the *No family formation* pattern are comparable to those of the *Late instability with moderate fertility* patterns. Overall, gender differences in predicted personal wealth levels are lowest in the *No family formation* pattern.

## Conclusion

This paper has adopted an innovative long-term approach to examine the extent to which the family life course is associated with wealth disparities at pre-retirement age (between ages 50 and 59) of Western Germans born between 1943 and 1967. Against the backdrop of increasingly heterogeneous family life courses and their relevance for the dynamics of social stratification, we proposed that departures from—or the stratified access to—a culturally and institutionally-supported family pattern of a stable marriage with (on average, two) children is associated with lower wealth at older age. We also proposed that the type of departure (regarding the occurrences, timings and ordering of typical family transitions) matters for wealth disparities at older age. Gender differences were also expected, given traditional gendered divisions in work and family roles. We tested these expectations using data from the German Socio-Economic Panel, and deployed sequence analysis to identify major family pathways. OLS regressions were used to predict respondents’ wealth ranks at ages 50–59.

Our results indicated that departure from the standard family trajectory was associated with substantially lower personal wealth for both men and women, after controlling for childhood characteristics that partly predict selection into family patterns and baseline wealth. However, women’s wealth ranks were substantially lower than those of men, in line with previous research on the gender wealth gap and the within-couple wealth gap (Grabka et al., 2015; Sierminska et al., 2010). In most cases, our results also supported our arguments about higher wealth penalties for greater deviation and lower penalties for moderate deviation from the standard pattern. A range of relevant empirical associations support this claim. First, small fertility-related variations from the socially normalised standard family life course were not linked to substantial wealth penalties for men or women in stable marriages. More substantial variation from the standard was exhibited by very high fertility (i.e. four or more children) within marriage and was linked to particularly high penalties for women but not men. Despite potentially high saving incentives and access to marital wealth premiums, childbearing-related opportunity costs for women rise with each child and accumulate over time. These child-related costs are not fully compensated by male partners as indicated by the substantial gap in predicted personal wealth levels of men and women in the very high fertility marriage pattern. This highlights mothers’ economic vulnerability even within a stable marriage. On the contrary, childlessness within marriage was associated with only negligible differences in personal wealth for women, and small declines for men. The absence of child-related career breaks for women results in longer time they can spend in the labour market and ultimately higher their wealth accumulation potentials. For men, low fertility or even the absence of fertility can be the result of meagre economic capacity or lower saving incentives. Second, patterns of marital instability were associated with low wealth ranks for men and women, reflecting the immediate costs and long-term wealth penalties of partnership dissolution. In addition, selection of financially disadvantaged couples into divorce and more complex family life courses likely matter. For women, highly complex family life courses that included marital dissolution but also aspects such as multi-partner fertility, early family transitions and unmarried childbearing were associated with the highest penalties. This highlights the interplay of different critical family life course aspect in the generation or amplification of inequalities. Wealth levels were not substantially penalised for remarried men who divorced early from their first marriage and had only one child. In comparison, women with a similar family life course experienced lasting disadvantages, potentially due childcare responsibilities while men may have had a substantial amount of time to recover financially, especially given the fact that child support from non-residential fathers is capped in Germany. Third, while the absence of marriage and childbearing over the life course can be considered a substantial deviation from the standard life course, this pattern was associated with only moderately though statistically significantly lower wealth for men but not women. Gender differences were also lowest among respondents in this family life course pattern. The fact that childless women do not incur child-related career disruptions might explain the small wealth difference.

Several of our study’s limitations are noteworthy. First, due to the mandatory nature of the German pay-as-you-go pension system, public pension entitlements are not collected in the SOEP. It may, however, be argued that such entitlements should be seen as an extension of working age income rather than wealth as German pension points cannot be liquidised, used as collateral, or passed on to next of kin (Sierminska et al., 2010). Second, survey questions about personal shares of jointly owned wealth may be ambiguous to respondents in terms of perceived or legal ownership. This may particularly be true for continuously married respondents. Nevertheless, it needs to be acknowledged that the data are currently unique in their provision of fully disaggregated wealth. Third, information on the time children spent in their parents’ household or with which parent they resided after divorce was not available retrospectively within the SOEP. Nevertheless, we argue that our approach provides crucial information on the relationship between parenthood and wealth, in intersection with marital histories. We argue that even if children do not reside in the same household as parents, child-related costs such as child allowance or financial transfers can influence economic decisions and saving incentives. Fourth, retrospective relationship information did not include information on cohabitation. For our study’ socio-historical context, this may be less problematic due to the low incidences of long-term cohabitation. However, for more recent cohorts the consideration of cohabitation within family life courses will be relevant to assess later life outcomes including wealth.

Despite these limitations, our study makes substantial contributions to the literature that addresses the links between family dynamics and economic wellbeing. We addressed entire family trajectories, from early adulthood to pre-retirement age, to extend and nuance our knowledge of the associations between earlier family behaviour and later economic wellbeing. While previous research has predominantly focused on marital histories and excluded the role of parenthood, our empirical exercise proved useful, combining marital and childbearing histories to highlight important and substantial disparities within groups of currently unmarried (i.e. ever divorced or never married) and currently married individuals depending on childbearing behaviours over the life course. Particularly for continuous marriage, we show relevant economic variation in older age depending on number of children, which was masked by previous research that focused solely on marital histories. Using comprehensive personal-level wealth data additionally provided a more thorough analysis of gender differences. Using per capita wealth—based on household-level wealth—obscures the fact that full financial access to all household resources is not always given. While income pooling and sharing has been shown to be less likely for childless marriages and within remarriage (Amuedo-Dorantes et al., 2011; Burgoyne & Morison, 1997), looking at personal wealth levels, our results show substantial gender wealth differences across continuously married and unmarried respondents at older ages. As gender differences are particularly prominent in groups characterized by above-average fertility within marriage, single parenthood, or divorce (with varying levels of fertility) the degree to which fathers and support systems compensate for the child-related depletion of women’s wealth accumulation is questionable.

Future research should scrutinise the intersection between marital and childbearing roles, including an assessment of the rate of wealth accumulation over time as individuals age or theoretical propositions and empirical tests of mechanisms that can explain the associations between specific family patterns and wealth. Such as analysis might also consider the additional interdependence between family and career transitions over the life course. Although we controlled for childhood characteristics that partly predict the stratified selection into family roles, we acknowledge that selection might also be due to socio-economic advantage achieved at later life stages. Therefore, empirical tests that elucidate the relevance of exposure to family roles and stratified selection into family roles for wealth disparities in older age are needed once more longitudinal wealth data become available. Additionally, it seems critical to consider how heterogeneity in family life course trajectories mediate the association between childhood characteristics including parental background and wealth in older age. As the standard family pattern is increasingly displaced by alternative patterns include arrangements such as stepfamilies or unmarried parents and new standards are established in line with ideas of a re-standardisation of the family domain (Huinink, 2013; Zimmermann & Konietzka, 2017), we can expect increasing social acceptance and political support at least for some these “new” family life course patterns in the near future. Given that, it is reasonable to expect that their association with wealth accumulation will also change. Nevertheless, some family pathways may remain or become more vulnerable. We should therefore continue monitoring the economic standing of diverse families.

## Supplementary Information

Below is the link to the electronic supplementary material.

Supplementary file1 (PDF 355 KB)

## Footnotes

1 We elaborate in more detail on the differences in family dynamics and wealth accumulation for West and East Germany in the supplementary material.

2 As widowhood is a rather uncommon event prior to retirement and thus also an uncommon occurrence in our cohort of interest (Statistisches Bundesamt, 2018), we focus on divorce as the reason for marital dissolution in the literature review.

3 The standard family life course (i.e. continuous marriage with two children) is also the most common pattern in our analytical sample.

4 Child-related costs can outweigh benefits if the number of children exceeds a financially manageable threshold for a particular household. To fully understand the child-related economic costs, it is relevant to additionally consider fertility levels alongside marital status. Manageable thresholds can be expected to be rather low for single parents as child-related direct and indirect costs are not fully covered jointly by both parents. In contrast, thresholds are higher for married parents due to the associated benefits of marriage.

5 Although the social acceptance of childbirth within cohabitation has increased, for the cohorts of interest in this study and the social context of West Germany, cohabitation was commonly seen as an undesirable family form for childbearing (Le Goff, 2002).

6 The supplementary material provides a brief description of the measurement of retrospective marital and childbearing information as well as the measurement of personal-level wealth.

7 While sequence data are not imputed, family cluster membership is used as an auxiliary variable in the imputation process. Table S.1 in the supplementary material provides an overview of variables used in the imputation processes and provides information on the number and share of missing data.

8 For men who entered the panel before 2000, fertility histories have been reconstructed using men’s female partners’ histories. We re-ran our sequence analysis and cluster analysis including that information. While the emerging family patterns are consistent with our main analysis, we argue that fertility cluster membership cannot properly be determined, particularly for non-stably partnered men.

9 This applies, for instance, to some SOEP refreshment samples.

10 As previous research has almost exclusively relied on household-level wealth data, we replicate our results using household-level wealth and provide detailed information on this measure including associated results in the supplementary material (section S.2.2 and Figures S.1 and S.2).

11 Less than two percent of respondents in this group are widows or widowers.

12 See section S.2.4 in the supplementary material for a justification of the use of a multi-channel sequence analysis.

13 This method is implemented within the *TraMineR* package using the *FUTURE* option.

14 We note that results do not vary substantively using a traditional OM distance (e.g. constant cost for substitution equal to 2, and for insertion and deletion equal to half of the cost of substitution).

15 For the visualisation we used relative frequency sequence plots (Fasang & Liao, 2014) and display one hundred (medoid) sequences sorted by the similarity of each cluster, which are representative of about every 3–7 sample sequences (left plot in the figures). To visually assess homogeneity across sequences in different regions of the cluster, the distance of the represented sequences to the representative (or medoid) sequence is also presented (right plot in the cluster); the larger the distance, the higher the heterogeneity across sequences.

## References

[CR1] Alessie R, Lusardi A, Aldershof T (1997). Income and wealth over the life cycle: Evidence from panel data. Review of Income and Wealth.

[CR2] Amuedo-Dorantes, C., Bonke, J., & Grossbard, S. (2011). Income pooling and household division of labor: Evidence from Danish couples. IZA Discussion Paper No. 5418. IZA, Bonn, Germany.

[CR3] Atkinson AB (1971). The distribution of wealth and the individual life-cycle. Oxford Economic Papers.

[CR4] Baizán P, Aassve A, Billari FC (2004). The interrelations between cohabitation, marriage and first birth in Germany and Sweden. Population and Environment.

[CR5] Bennett F (2013). Researching within-household distribution: Overview, developments, debates, and methodological challenges. Journal of Marriage and Family.

[CR6] Bernardi, F., Boertien, D., & Geven, K. (2019). Childhood family structure and the accumulation of wealth across the life course. *Journal of Marriage and Family, 81*(1), 230–247. 10.1111/jomf.12523

[CR7] Bessière C (2019). Reversed accounting: Legal professionals, families and the gender wealth gap in France. Socio-Economic Review, Online First.

[CR8] Bradbury B, Ben-Arieh A, Casas F, Frønes I, Korbin JE (2011). Child costs. Handbook of child well-being.

[CR9] Brückner H, Mayer KU (2005). De-standardization of the life course: What it might mean? And if it means anything, whether it actually took place?. Advances in Life Course Research.

[CR10] Budig MJ, England P (2001). The wage penalty for motherhood. American Sociological Review.

[CR11] Burgoyne CB, Morison V (1997). Money in remarriage: Keeping things simple–and separate. The Sociological Review.

[CR12] Chang ML (2010). Shortchanged: Why women have less wealth and what can be done about it.

[CR13] Dannefer D (2003). Cumulative advantage/disadvantage and the life course: Cross-fertilizing age and social science theory. The Journals of Gerontology: Series B.

[CR14] Deutsche Rentenversicherung Bund (2018). Rentenversicherung in Zeitreihen [Time series of pension insurances]. DRV-Schriften 22.

[CR15] Eads A, Tach L (2016). Wealth and inequality in the stability of romantic relationships. RSF: The Russell Sage Foundation Journal of the Social Sciences.

[CR16] Ebbinghaus B (2015). The privatization and marketization of pensions in Europe: A double transformation facing the crisis. European Policy Analysis.

[CR17] Eickmeyer KJ, Manning WD, Brown SL (2019). What’s mine is ours? Income pooling in American families. Journal of Marriage and Family.

[CR18] Fasang AE, Liao TF (2014). Visualizing sequences in the social sciences. Sociological Methods and Research.

[CR19] Gabadinho A, Ritschard G, Studer M, Müller N (2008). Mining sequence data in R with the TraMineR package: A user’s guide.

[CR20] Gauthier J-A, Widmer ED, Bucher P, Notredame C (2010). Multichannel sequence analysis applied to social science data. Sociological Methodology.

[CR21] Gibson-Davis CM, Edin K, McLanahan S (2005). High hopes but even higher expectations: The retreat from marriage among low-income couples. Journal of Marriage and Family.

[CR22] Goebel, J., Grabka, M. M., Liebig, S., Kroh, M., Richter, D., Schröder, C., & Schupp, J. (2019). The German Socio-Economic Panel (SOEP). *Jahrbücher für Nationalökonomie und Statistik,**239*(2), 345–360. 10.1515/jbnst-2018-0022

[CR23] Grabka MM, Marcus J, Sierminska E (2015). Wealth distribution within couples. Review of Economics of the Household.

[CR24] Grabka, M. M., & Westermeier, C. (2015). Editing and multiple imputation of item non-response in the wealth module of the German Socio-Economic Panel. SOEP Survey Papers, Series C–Data Documentation, No. 272. DIW, Berlin, Germany.

[CR25] Hakim C (1992). Explaining trends in occupational segregation: The measurement, causes, and consequences of the sexual division of labour. European Sociological Review.

[CR26] Hakovirta M, Meyer DR, Skinner C (2019). Does paying child support impoverish fathers in the United States, Finland, and the United Kingdom?. Children and Youth Services Review.

[CR27] Halpern-Manners A, Warren JR, Raymo JM, Nicholson DA (2015). The impact of work and family life histories on economic well-being at older ages. Social Forces.

[CR28] Huinink J, Neyer G, Andersson G, Kulu H, Bernardi L, Bühler C (2013). De-standardisation or changing life course patterns? Transition to adulthood from a demographic perspective. The demography of Europe.

[CR29] Hurd MD, Guiso L, Haliassos M, Jappelli T (2002). Portfolio holdings of the elderly. Household portfolios.

[CR30] Jalovaara M, Fasang AE (2020). Family life courses, gender, and mid-life earnings. European Sociological Review.

[CR31] Joseph R, Rowlingson K (2012). Her house, his pension? The division of assets among (ex-)couples and the role of policy. Social Policy and Society.

[CR32] Kapelle N, Baxter J (2021). Marital dissolution and personal wealth: Examining gendered trends across the dissolution process. Journal of Marriage and Family.

[CR33] Kapelle N, Lersch PM (2020). The accumulation of wealth in marriage: Over-time change and within-couple inequalities. European Sociological Review.

[CR34] Killewald A, Pfeffer FT, Schachner JN (2017). Wealth inequality and accumulation. Annual Review of Sociology.

[CR35] Kohli M (2007). The institutionalization of the life course: Looking back to look ahead. Research in Human Development.

[CR36] Le Goff J-M (2002). Cohabiting unions in France and West Germany: Transitions to first birth and first marriage. Demographic Research.

[CR37] Leopold T, Schneider T (2011). Family events and the timing of intergenerational transfers. Social Forces.

[CR38] Lersch PM (2017). The marriage wealth premium revisited: Gender disparities and within-individual changes in personal wealth in Germany. Demography.

[CR39] Lersch PM, Jacob M, Hank K (2017). Parenthood, gender, and personal wealth. European Sociological Review.

[CR40] Lillard LA (1977). Inequality: Earnings vs human wealth. The American Economic Review.

[CR41] Lusardi A, Cossa R, Krupka EL (2001). Savings of young parents. The Journal of Human Resources.

[CR42] Madero-Cabib I, Fasang AE (2016). Gendered work–family life courses and financial well-being in retirement. Advances in Life Course Research.

[CR43] Manning WD, Stewart SD, Smock PJ (2003). The complexity of fathers’ parenting responsibilities and involvement with nonresident children. Journal of Family Issues.

[CR44] Maroto M (2018). Saving, sharing, or spending? The wealth consequences of raising children. Demography.

[CR45] Mayer KU (2004). Whose lives? How history, societies, and institutions define and shape life courses. Research in Human Development.

[CR46] McLanahan S, Percheski C (2008). Family structure and the reproduction of inequalities. Annual Review of Sociology.

[CR47] Milligan K (2005). Life-cycle asset accumulation and allocation in Canada. Canadian Journal of Economics.

[CR48] Modigliani F (1988). The role of intergenerational transfers and life cycle saving in the accumulation of wealth. The Journal of Economic Perspectives.

[CR49] Montalto CP, Yuh Y, Hanna S (2000). Determinants of planned retirement age. Financial Services Review.

[CR50] Muller JS, Hiekel N, Liefbroer AC (2020). The long-term costs of family trajectories: Women’s later-life employment and earnings across Europe. Demography.

[CR51] O’Rand AM (1996). The precious and the precocious: Understanding cumulative disadvantage and cumulative advantage over the life course. The Gerontologist.

[CR52] OECD (2013). OECD framework for statistics on the distribution of household income, consumption and wealth.

[CR53] Oppenheimer VK (1988). A theory of marriage timing. American Journal of Sociology.

[CR54] Perales F (2013). Occupational sex-segregation, specialized human capital and wages: Evidence from Britain. Work, Employment and Society.

[CR55] Pfeffer FT, Killewald A (2017). Generations of advantage: Multigenerational correlations in family wealth. Social Forces.

[CR56] Pfeffer FT, Schoeni RF (2016). How wealth inequality shapes our future. RSF: The Russell Sage Foundation Journal of the Social Sciences.

[CR57] Ponomarenko V (2017). Wealth accumulation over the life course: The role of disadvantages across the employment history.

[CR58] Rubin DB (1987). Multiple imputation for nonresponse in surveys.

[CR59] Schneider D (2011). Wealth and the marital divide. American Journal of Sociology.

[CR60] Seeleib-Kaiser M (2016). The end of the conservative German welfare state model. Social Policy and Administration.

[CR61] Sierminska E, Frick JR, Grabka MM (2010). Examining the gender wealth gap. Oxford Economic Papers.

[CR62] Statistisches Bundesamt (2018). Datenreport 2018–Kapitel 1: Bevölkerung und Demografie [Data report 2018–chapter 1: Population and demography].

[CR63] Studer M, Ritschard G (2016). What matters in differences between life trajectories: A comparative review of sequence dissimilarity measures. Journal of the Royal Statistical Society: Series A.

[CR64] Trappe H, Pollmann-Schult M, Schmitt C (2015). The rise and decline of the male breadwinner model: Institutional underpinnings and future expectations. European Sociological Review.

[CR65] Ulker A (2008). Wealth holdings and portfolio allocation of the elderly: The role of marital history. Journal of Family and Economic Issues.

[CR66] Upchurch DM, Lillard LA, Panis CWA (2002). Nonmarital childbearing: Influences of education, marriage, and fertility. Demography.

[CR67] Wilmoth J, Koso G (2002). Does marital history matter? Marital status and wealth outcomes among preretirement adults. Journal of Marriage and Family.

[CR68] Xie Y, Raymo JM, Goyette K, Thornton A (2003). Economic potential and entry into marriage and cohabitation. Demography.

[CR69] Zimmermann O, Konietzka D (2017). Social disparities in destandardization: Changing family life course patterns in seven European countries. European Sociological Review.

[CR70] Zissimopoulos JM, Karney BR, Rauer AJ (2015). Marriage and economic well being at older ages. Review of Economics of the Household.

