# Supplementary material for: Heterogeneity in Family Life Course Patterns and Intra-Cohort Wealth Disparities in Late Working Age
Source: Eur J Popul. 2021 Dec 23;38(1):59–92. doi: 10.1007/s10680-021-09601-4 (PMC8924336; doi:10.1007/s10680-021-09601-4)
Supplement: Supplementary file 1 — Supplementary file1 (PDF 355 KB) [file 10680_2021_9601_MOESM1_ESM.pdf]

Supplementary material

Heterogeneity in family life course patterns and intra-cohort wealth disparities in late working age

S.1 The division of Germany and its influence on the family and economic standing

Family life courses and the accumulation of wealth are embedded in the historical social and political context (Mayer, 2004). The majority of family life courses and financial decisions of our German cohorts of interest were made during the division of Germany between 1955 and 1990. The Federal Republic of Germany (FRG) in the West and the German Democratic Republic (GDR) in the East had substantially different economic and welfare systems, which steered decisions about family transitions and within-couple work arrangements but also determined the capacity and perceived need to accumulate wealth.

In the FRG, policies encouraged a male-breadwinner/female-homemaker specialisation (Brückner, 2004), which lead to a decreased labour market attachment of women and to a postponement of marriage and childbearing until education was completed and careers established. The GDR promoted population growth through pronatalist family policies that facilitated the compatibility of family life and work for all social strata (Huinink et al., 1995; Kreyenfeld, 2004). In combination with officially zero unemployment, dual earner couples were the norm in the GDR. Access to the rental market and other benefits was further provided based on a need scheme which placed married couples, parents, or divorcees above childless singles. These policies resulted in a younger age at first marriage and childbirth for GDR cohorts compared the FRG cohorts. It also facilitated higher rates of out of wedlock births and divorces in the former East compared to the former Western part of Germany. For GDR cohorts born in the late 1950s and afterwards, the age at first marriage was higher than age at first childbirth (Huinink, 1997). Hence, while the former FRG incentivised the adherence to the 'traditional' family model, structures in the former GDR enabled individuals to tread new paths (Huinink, Kreyenfeld, & Trappe, 2012; Klüsener & Goldstein, 2016; Kreyenfeld, Konietzka, & Walke, 2011). Nevertheless, the nuclear family was the most popular family type in either states (Dennis, 1998). According to the last GDR census of 1981, 56.4 percent of families were nuclear families of which over 90 percent had one to two children (Rueschemeyer, 1988). Similarly, recent results from Van Winkle (2018) showed that family complexity in the two German countries were surprisingly similar.

Capabilities to accumulate private wealth were steered differently in the two systems. While the social market economy of the FGR incentivised private savings and investments, the centrally planned economy in the GDR substantially limited private investments and assets ownership. Starting in 1945, the GDR additionally expropriated a large proportion of private

property and business assets. As a result, only 41 percent of GDR housing property was privately owned by the end of the 1980s compared to 91 percent in the FGR. Due to the lack of investment options in the GDR, most private wealth was therefore accumulated in savings accounts. At reunification, expropriated property was returned to their rightful owners, GDR Mark converted favourable to Deutsche Mark and large economic incentives were put in place to aid former GDR residents in their wealth accumulation (Hauser et al., 1996). Nevertheless, even decades after reunification wealth disparities between eastern and western Germany remain substantial particularly amongst the older population (Grabka & Westermeier, 2014).

S.2 Additional description of data and method

S.2.1 SOEP retrospective family histories data

The SOEP provides retrospective marital and fertility information within the datasets BIOMARSY[1] and BIOBIRTH. Retrospective information is collected using a biographical questionnaire, which is administered once within one of the first years after panel entry. An exception is men's fertility history data, which have only been collected for men who entered the SOEP in 2000 or later. Retrospective datasets are updated annually using information provided within the personal questionnaire regarding the current family status and family events that may have occurred since 1st January of the previous year. Detailed information on retrospective data is available in Goebel (2017).

S.2.2 Additional information on personal-level SOEP wealth data

Whereas other panel studies commonly measure wealth at the household level and one household member provides information on the financial standing of the entire household, within the SOEP, wealth information is measured at the individual level. This means that each household member over 16 years of age is questioned about their personal and potentially shared assets and liabilities. The SOEP is thus currently the only household panel study that provides comprehensive personal-level wealth measures over four waves.

Wealth data collection follows several steps. First, a filter question (yes/no) is asked to assess whether the respondent personally holds a certain type of assets or liability. Second, if respondents answer in the affirmative, they are asked to provide the total value. Third, a second filter question (yes/no) is posed to assess whether those assets and liabilities are held jointly. This is only done for wealth components that can theoretically be owned jointly (e.g. housing equity). Fourth, if respondents affirm joint ownership, they are asked about their personal share in percentage points.

Using the total metric value of the wealth component and personal share, the SOEP team calculates the value of personally owned assets and liabilities. Based on all household members' personal wealth, the SOEP team further aggregates personal-level wealth to the household-level, so that SOEP users are provided with both personal-level and household-level wealth measurements (Grabka & Westermeier, 2015).

[1] Additionally, the SOEP provides monthly retrospective marital histories within the dataset BIOMARSM, which was however not used within the current study.

As previous research has almost exclusively relied on household-level wealth data in the analysis of wealth at older ages, we re-run our analyses using total per capita net wealth. To generate this measure, we use household-level wealth data, which in the SOEP is personal-level wealth aggregated to the household. We divide household-level wealth by the number of adults living in the household to obtain the per capita measure. Results of this supplementary analysis are provided in Figure S.1. and S.2. in this supplementary material. Although the general directions of the association of interest are in line with our main results, due to the nature of the measure and the neglect of within-couple wealth differences, gender differences are substantially reduced for the per capita measure.

S.2.3 Confounders and additional measures used within the descriptive and multivariate analyses

A range of baseline confounders are included as control variables in the regression analyses, as they partially predict both selection into certain family pathways and base-level wealth. These include: a dummy for migration background to indicate whether respondents or their parents had immigrated to Germany; a categorical measure of the number of siblings (none (ref.), 1 sibling, 2 siblings, 3 or more siblings); a continuous measure of parents' occupational status defined by the Standard International Occupational Prestige Scale (SIOPS); and a categorical measure of parents' highest education level (low (ref.), intermediate, high). Additionally, the regression models control for respondents' age as a continuous measure to capture maturation effects and account for age related wealth differences within our sample; respondents' birth cohorts (1943-1950 (ref.), 1951-1958, 1959-1967) to consider cohort effects; and marital status changes between ages 50 and 59 (depending on age at last observation) by including three dummy variables that capture the entry into marriage, or marital dissolution either through separation and divorce or through widowhood.

While the present paper does not aim to explain the specific mechanisms of wealth accumulation associated with different family trajectories, we partially address the resource accumulation potential of major family trajectories within our descriptive analyses. For this, we use the following human capital trajectory measures separately for men and women: respondents' highest level of education (low, intermediate, high), number of years of employment, number of unemployment episodes, and the mode of the Standard International Occupational Prestige Scale (SIOPS) score.

S.2.4 Justifications for the use of a multi-channel sequence analysis

Sequence analysis applications examining interdependencies across (multiple) life domains have typically relied on two strategies: combining states across sequences of two or more domains, or performing domain-specific sequence analysis and averaging the resulting pairwise dissimilarities. Instead, we chose MCSA because it enables us to consider all possible interactions across domain-specific states (i.e. using a full set of combined states was not feasible given sample limitations) and properly acknowledging relevant cross-domain interdependence (often not accomplished when averaging domain-specific distances).

Key requirements for MCSA applications are that the study domains should be interdependent, and the domain-based dissimilarities should be associated with MCSA dissimilarities. Deploying the approach proposed by Piccarreta (2017), we find that the marital and fertility domains are associated, though moderately (Cronbach's alpha value of 0.34 based on Mantel's correlations between domain-specific pairwise dissimilarities), and that the MCSA pairwise dissimilarities capture the relevant heterogeneity in the marital and fertility domains (Pearson correlation over 0.65 between domain-joint and domain-specific pairwise dissimilarities; and R-square of 0.58 or the share of variance of the dissimilarities in the domain-joint pairwise dissimilarities that is reproduced by the domain-specific pairwise dissimilarities).

S.2.5 Assessment of cluster quality of 11-cluster solution

The overall average silhouette width (ASW) of the 11-cluster solution is 0.34, which indicates that the homogeneity of the clusters is moderate. It is worth noting that the two remarriage clusters (i.e. remarriage with low fertility and remarriage with high complexity) display ASW below .20 suggesting low within-cluster homogeneity. This was expected since these clusters combine sets of complex sequences that vary on the timing and duration of state episodes, but there are relevant common sequencings, including union dissolution, repartnering and multiple fertility episodes. See sequence-specific and cluster-specific ASW as well as cluster-specific sample distributions in Figure S.4. and Table S.2., respectively.

S.3 Additional tables and figures

Table S.1. Variables used for the multiple imputation. Number and percentages of imputed missing data.

Variable category	Variable	2002		2007		2012		2017	
		Missing values	Share of missing values	Missing values	Share of missing values	Missing values	Share of missing values	Missing values	Share of missing values
Wealth	Personal net wealth (rank)*	none (SOEP imputed data used)							
Basic demographics	Gender*	none							
	Age*	none							
	Migration background*	none							
	SOEP sample	none							
	Federal state	none							
	Living area	0	0.00	44	2.16	40	1.65	25	0.85
Family	Family pattern*	none							
	Divorce after age 50*	none							
	Marriage after age 50*	none							
	Widowhood after age 50*	none							
Family of origin	Parental education*	155	7.72	136	6.68	86	3.55	99	3.37
	Parental SIOPS*	367	18.29	306	15.03	255	10.53	233	7.93
	Number of siblings*	0	0.00	0	0.00	1	0.04	20	0.68
Human capital	Full-time employment experience	1	0.06	1	0.06	1	0.05	4	0.16
	Number of unemployment spells	none							
	SIOPS mode	356	17.74	229	11.25	228	9.42	195	6.64
	Highest level of education	1	0.05	4	0.20	3	0.12	10	0.34

Notes: Data are from Socio-Economic Panel Survey v34 (2002, 2007, 2012, 2017)
*Variables used in regression analyses. Variables without a * are used as auxiliary variables for the imputation and to describe the family typology descriptively.

Figure S.1. Predicted per capita wealth rank of men and women aged 50 to 59 in the standard family pattern and the non-standard family pattern based on multivariable OLS regression models.

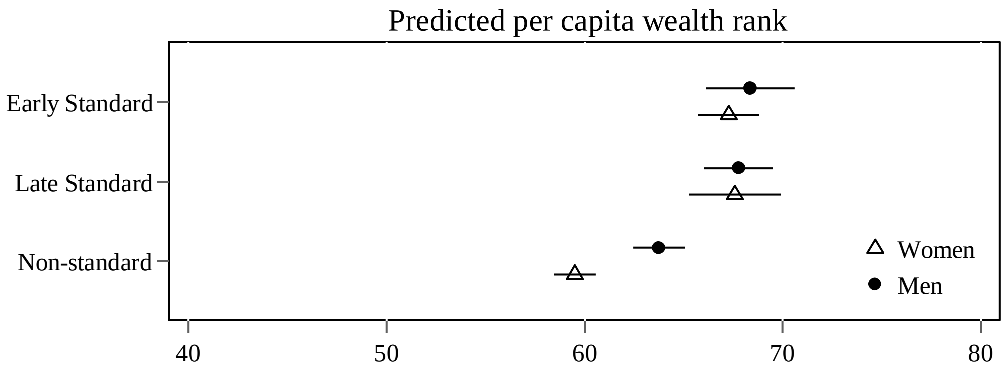

Notes: Whiskers indicate 95% confidence intervals. Data are from the Socio-Economic Panel Survey v34 (2002, 2007, 2012, 2017; unweighted; multiply imputed). Models include control variables for age, migration background, birth cohort, number of siblings, parental education, parental occupational prestige, marital events after the age of 50 (marriage, divorce, widowhood).

Figure S.2. Predicted per capita wealth rank of men and women aged 50 to 59 across the diversity of family patterns based on multivariable OLS regression models.

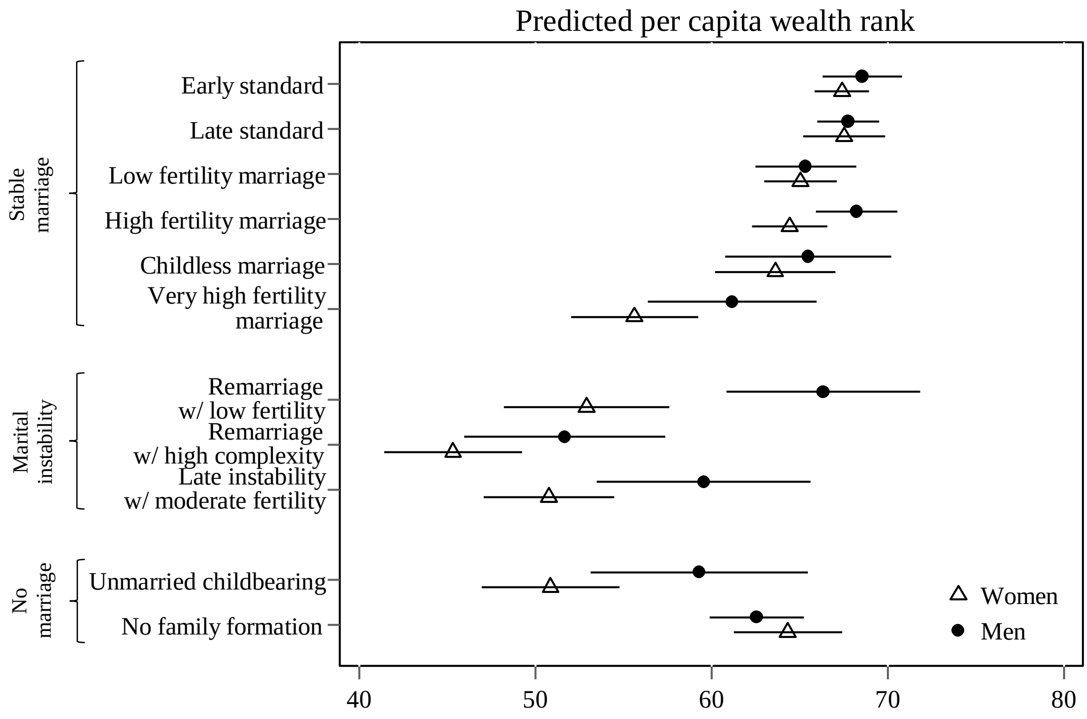

Notes: Whiskers indicate 95% confidence intervals. Data are from the Socio-Economic Panel Survey v34 (2002, 2007, 2012, 2017; unweighted; multiply imputed). Models include control variables for age, migration background, birth cohort, number of siblings, parental education, parental occupational prestige, marital events after the age of 50 (marriage, divorce, widowhood).

Figure S.3. Cluster cut-off criteria

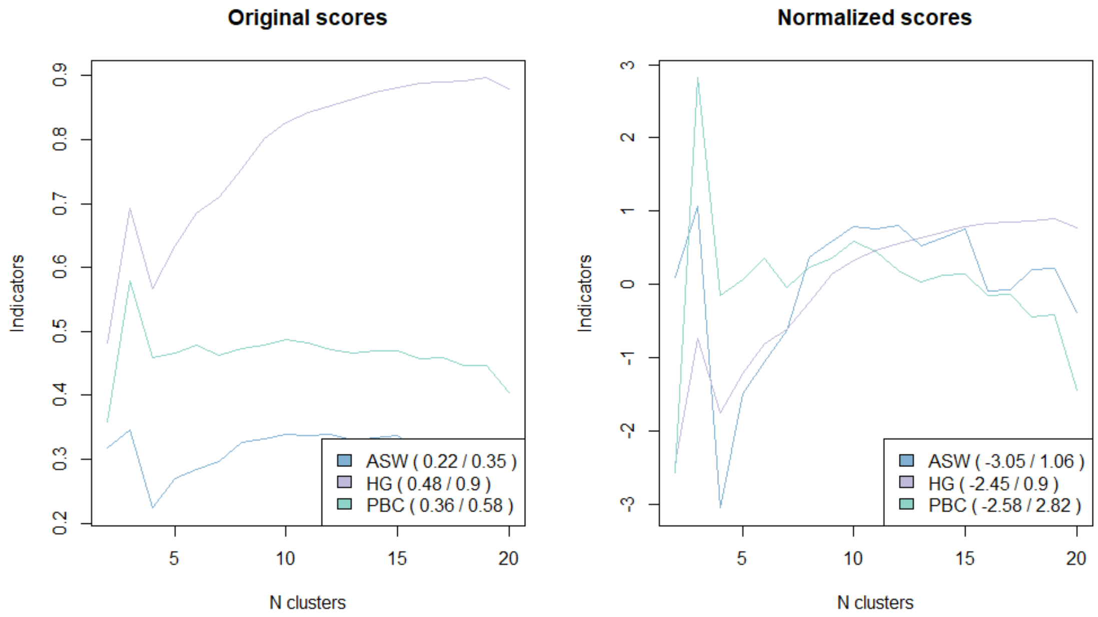

Notes: ASW-Average silhouette width; HG- Hubert’s Gamma; PBC-Point Biserial Correlation (see Studer (2013) for definitions).

Figure S.4. Cluster-specific Average Silhouette Widths

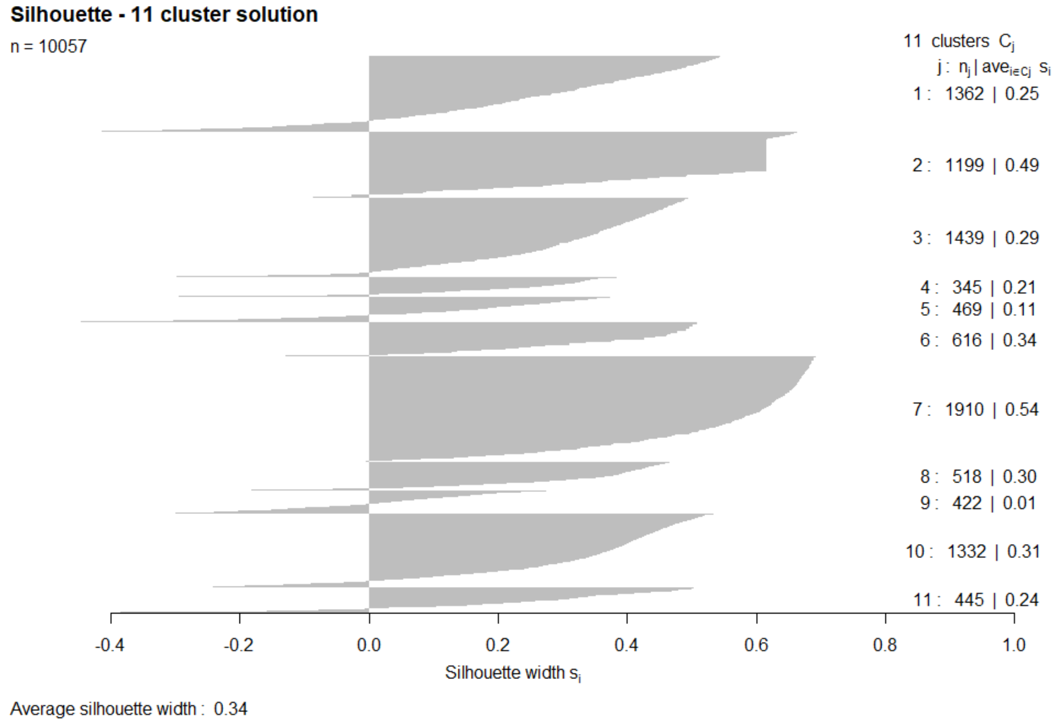

Notes: j = cluster index; n = sample size (of cluster j); S = average Silhouette (of cluster j); Cluster j indexes: (1) Late standard, (2) No family formation, (3) Low fertility marriage, (4) Remarriage with low fertility, (5) Late instability with moderate fertility, (6) Childless marriage, (7) Early standard, (8) Very high fertility marriage, (9) Remarriage with high complexity, (10) High fertility marriage, (11) Non-marital childbearing.

Table S.2. Case numbers across the eleven family pathways.

Family patterns	Men		Women		Total	
	N	%	N	%	N	%
Early standard	365	12.92	920	22.01	1,285	18.35
Late standard	593	21.00	413	9.88	1,006	14.36
Stable marriage w/ 1 child	358	12.68	618	14.78	976	13.93
Stable marriage w/ 3 children	374	13.24	560	13.40	934	13.34
Childless stable marriage	158	5.59	261	6.24	419	5.98
Stable marriage w/ 4+ children	120	4.25	227	5.43	347	4.95
Remarriage w/ low fertility	91	3.22	160	3.83	251	3.58
Remarriage w/ high complexity	90	3.19	219	5.24	309	4.41
Late marital instability w/ moderate fertility	89	3.15	245	5.86	334	4.77
Unmarried childbearing	91	3.22	214	5.12	305	4.35
No family formation	495	17.53	343	8.21	838	11.96
**Total**	**2,824**	**100.00**	**4,180**	**100.00**	**7,004**	**100.00**

Notes: Data are from Socio-Economic Panel Survey v34 (2002, 2007, 2012, 2017)

Table S.3. Summary indicators of major family patterns using non-imputed data

	Family patterns											Total
	Stable marriage						Marital instability			No marriage		
	Early standard	Late standard	Low fertility marriage	High fertility marriage	Childless marriage	Very high fertility marriage	Re-marriage w/ low fertility	Re-marriage w/ high complexity	Late instability w/ mode-rate fertility	Un-married child-bearing	No family formation	
	mean/(SE)	mean/(SE)	mean/(SE)	mean/(SE)	mean/(SE)	mean/(SE)	mean/(SE)	mean/(SE)	mean/(SE)	mean/(SE)	mean/(SE)	mean/(SE)
**Wealth levels**												
Personal net wealth	181.27<br>(284.34)	221.16<br>(336.86)	182.64<br>(254.54)	221.51<br>(411.84)	205.68<br>(288.92)	172.14<br>(383.92)	124.84<br>(219.75)	143.14<br>(397.13)	161.73<br>(332.90)	119.45<br>(265.10)	188.95<br>(375.49)	187.06<br>(331.12)
**Basic demographics**												
Female	0.69	0.38	0.62	0.56	0.57	0.54	0.64	0.69	0.72	0.72	0.41	0.57
Migration background	0.07	0.09	0.06	0.08	0.06	0.11	0.05	0.10	0.08	0.14	0.06	0.08
Cohort												
1943-1950	0.30	0.15	0.24	0.17	0.21	0.17	0.24	0.15	0.18	0.05	0.16	0.20
1951-1958	0.44	0.30	0.38	0.35	0.41	0.38	0.39	0.42	0.32	0.19	0.36	0.37
1959-1966	0.27	0.55	0.37	0.48	0.38	0.46	0.37	0.42	0.50	0.76	0.49	0.44
Number of siblings	2.09<br>(1.86)	1.98<br>(1.66)	1.81<br>(1.50)	2.20<br>(1.80)	1.73<br>(1.68)	2.53<br>(2.05)	2.05<br>(1.72)	2.45<br>(1.99)	1.89<br>(1.59)	2.04<br>(1.71)	1.84<br>(1.57)	2.02<br>(1.73)
Parental education												
Low	0.18	0.11	0.14	0.17	0.09	0.19	0.21	0.16	0.15	0.13	0.09	0.14
Middle	0.76	0.71	0.77	0.70	0.79	0.67	0.73	0.75	0.75	0.74	0.77	0.74
High	0.05	0.18	0.08	0.12	0.12	0.14	0.06	0.08	0.10	0.13	0.13	0.11
Parental occupational prestige	39.66	44.91	41.40	42.31	43.13	43.50	39.47	41.00	41.61	42.45	44.41	42.28
**Family pattern until age 50**												
Age at first birth	24.67<br>(3.32)	32.86<br>(3.65)	29.80<br>(5.60)	26.75<br>(4.49)	0.78<br>(5.87)	25.42<br>(4.30)	28.80<br>(7.23)	23.54<br>(4.61)	25.89<br>(4.25)	30.58<br>(6.19)	9.35<br>(17.32)	23.96<br>(11.69)
Number of children	2.00<br>(0.05)	2.20<br>(0.58)	1.02<br>(0.18)	3.01<br>(0.09)	0.03<br>(0.28)	4.49<br>(0.85)	1.27<br>(0.62)	3.12<br>(0.96)	2.08<br>(0.31)	1.50<br>(0.79)	0.38<br>(0.80)	1.82<br>(1.19)
Unmarried childbearing	0.20	0.27	0.18	0.26	0.01	0.35	0.46	0.57	0.42	1.00	0.11	0.27
Multi-partner childbearing	0.00	0.01	0.01	0.01	0.00	0.03	0.11	0.51	0.14	0.00	0.00	0.04
Age at first	23.58	31.89	27.19	26.08	26.83	25.39	23.80	22.27	24.82	10.79	22.52	25.27

marriage	(2.88)	(4.10)	(5.53)	(4.28)	(4.39)	(3.49)	(5.03)	(3.58)	(4.12)	(19.02)	(20.65)	(10.05)
Ever married	1.00	1.00	1.00	1.00	1.00	1.00	1.00	1.00	1.00	0.25	0.55	0.91
Ever divorced	0.04	0.09	0.24	0.13	0.41	0.14	0.98	0.93	0.86	0.03	0.03	0.23
Ever remarried	0.00	0.00	0.06	0.02	0.16	0.03	0.78	0.84	0.33	0.00	0.01	0.11
**Human capital - men**												
Education												
Low	0.03	0.03	0.04	0.07	0.04	0.11	0.03	0.13	0.01	0.07	0.07	0.05
Middle	0.60	0.37	0.50	0.35	0.50	0.36	0.66	0.55	0.58	0.51	0.47	0.46
High	0.37	0.60	0.45	0.58	0.46	0.54	0.31	0.30	0.41	0.42	0.46	0.49
Full-time	32.25	28.08	30.35	29.46	30.29	29.14	31.50	29.46	30.90	28.43	26.96	29.31
employment years	(4.73)	(6.22)	(6.38)	(6.37)	(6.24)	(6.40)	(7.55)	(6.76)	(4.98)	(6.54)	(7.72)	(6.62)
Non-/Un-	0.31	0.47	0.51	0.44	0.56	0.48	0.75	0.91	0.65	1.04	0.77	0.56
employment episodes	(0.67)	(0.85)	(0.84)	(0.82)	(0.86)	(1.01)	(1.23)	(1.26)	(1.01)	(1.33)	(1.13)	(0.95)
Occupational prestige mode	46.96	50.83	49.11	50.59	48.81	50.52	46.48	43.68	45.80	44.69	48.19	48.80
**Human capital - women**												
Education												
Low	0.11	0.04	0.05	0.09	0.04	0.17	0.10	0.24	0.09	0.12	0.04	0.09
Middle	0.68	0.43	0.67	0.55	0.54	0.48	0.72	0.63	0.67	0.51	0.41	0.58
High	0.21	0.53	0.29	0.36	0.42	0.33	0.18	0.13	0.24	0.37	0.55	0.33
Full-time	11.21	12.67	16.77	10.32	26.66	7.43	16.98	12.75	14.42	15.94	24.38	14.84
employment years	(9.72)	(8.29)	(10.41)	(8.37)	(9.00)	(7.62)	(9.68)	(9.40)	(8.84)	(9.28)	(9.59)	(10.58)
Non-/Un-	2.08	2.16	1.75	2.32	1.38	2.43	2.35	2.59	2.56	2.14	1.39	2.06
employment episodes	(1.30)	(1.34)	(1.35)	(1.30)	(1.45)	(1.31)	(1.45)	(1.24)	(1.31)	(1.36)	(1.44)	(1.39)
Occupational prestige mode	41.61	49.00	45.06	44.47	48.60	41.95	44.34	39.76	41.20	44.84	50.56	44.62
**Observations**	813	709	642	589	286	206	171	226	250	198	580	4670
**Individuals**	627	580	522	474	223	171	135	175	201	173	470	3751
**% respondents**	16.72	15.46	13.92	12.64	5.95	4.56	3.60	4.67	5.36	4.61	12.53	100.00

Notes: Data are from Socio-Economic Panel Survey v34 (2002, 2007, 2012, 2017); non-imputed, unweighted.

Table S.4. Multivariate regression models of personal net wealth (rank transformed) with gender interactions

	Standard vs non-standard B/(SE)	Full family heterogeneity B/(SE)
Family pattern (Ref.: Early standard)		
Non-standard	-7.06***	
	(1.56)	
Late standard	-1.41	
	(1.98)	
Female (Ref.: Male)	-7.29***	-7.30***
	(1.54)	(1.65)
Gender interaction		
Non-standard X Female	-0.03	
	(1.73)	
Late standard X Female	4.07	
	(2.33)	
Family patterns (Ref.: Early standard)		
Late standard		-1.67
		(1.99)
Low fertility marriage		-5.36*
		(2.13)
High fertility marriage		1.64
		(1.99)
Childless marriage		-7.03*
		(2.81)
Very high fertility marriage		-4.57
		(3.07)
Remarriage w/ low fertility		-8.91*
		(3.68)
Remarriage w/ high complexity		-18.40***
		(3.58)
Late instability w/ moderate fertility		-12.19***
		(3.54)
Unmarried childbearing		-17.34***
		(4.01)
No family formation		-11.37***
		(2.03)
Gender interaction		
Late standard X Female		4.05
		(2.45)
Low fertility marriage X Female		3.33
		(2.53)
High fertility marriage X Female		-3.34
		(2.60)
Childless marriage X Female		2.97
		(3.56)
Very high fertility marriage X Female		-3.51
		(3.97)
Remarriage w/ low fertility X Female		-3.46
		(4.77)
Remarriage w/ high complexity X Female		-2.48
		(4.48)
Late instability w/ moderate fertility X Female		-5.39
		(4.26)
Unmarried childbearing X Female		-0.04
		(4.48)

No family formation X Female		7.78**
		(2.56)
Age	0.42***	0.44***
	(0.12)	(0.11)
Migration background	-14.57***	-14.58***
	(1.51)	(1.47)
Birth cohort (Ref.: 1943-1950)		
1951-1958	-0.61	-0.29
	(0.93)	(0.90)
1959-1966	-4.11***	-2.98**
	(1.07)	(1.01)
Number of siblings (Ref.: None)		
1	-0.79	-0.71
	(1.13)	(1.10)
2	-3.05*	-3.18*
	(1.26)	(1.24)
3 or more	-7.38***	-7.22***
	(1.23)	(1.20)
Parental educational level (Ref.: Low)		
Intermediate	4.86***	4.82***
	(1.16)	(1.14)
High	9.12***	9.04***
	(1.77)	(1.77)
Parental occupational prestige	0.28***	0.28***
	(0.04)	(0.04)
Ever married between age 50 to 59	-3.96*	-2.11
	(2.00)	(1.95)
Ever divorced between age 50 to 59	-10.88***	-11.03***
	(1.99)	(2.03)
Ever widowed between age 50 to 59	-4.57	-4.17
	(2.67)	(2.62)
Constant	41.16***	39.82***
	(6.81)	(6.56)
N Observations	9402	9402
N Individuals	7004	7004

Notes: Data are from Socio-Economic Panel Survey v34 (2002, 2007, 2012, 2017); imputed, unweighted.
* p<.05, ** p<.01, *** p<.001

References

Brückner, H. (2004). *Gender inequality in the life sourse: Social change and stability in West Germany 1975–1995*. Somerset, NJ: Transaction Publishers.

Dennis, M. (1998). The East German family: Change and continuity. *German Politics, 7*(3), 83-100. doi:10.1080/09644009808404528

Goebel, J. (2017). *SOEP-Core v32: Documentation on biography and life history data*. Retrieved from Berlin, Germany:

Grabka, M. M., & Westermeier, C. (2014). Persistently high wealth inequality in Germany. *DIW Economic Bulletin, 6*, 3-15.

Grabka, M. M., & Westermeier, C. (2015). *Editing and multiple imputation of item non-response in the wealth module of the German Socio-Economic Panel*. SOEP Survey Papers, Series C - Data Documentation, No. 272. DIW, Berlin, Germany.

Hauser, R., Glatzer, W., Hradil, S., Kleinhenz, G., Olk, T., & Pankoke, E. (1996). *Ungleichheit und Sozialpolitik [Inequality and social policy]*. Opladen, Germany: Leske + Budrich.

Huinink, J. (1997). Vergleichende Familienforschung: Ehe und Familie in der ehemaligen DDR und der Bundesrepublik Deutschland [Comparative family studies: Marriage and family in the former GDR and FRG]. In L. A. Vaskovics (Ed.), *Familienleitbilder und Familienrealitäten* (pp. 308-325). Wiesbaden: VS Verlag für Sozialwissenschaften.

Huinink, J., Kreyenfeld, M., & Trappe, H. (2012). Familie und Partnerschaft in Ost- und Westdeutschland: Ähnlich und doch immer noch anders [Family and partnership in Eastern and Western Germany: Similar but still different]. *Journal of Family Research, Special Issue*(9), 5-24.

Huinink, J., Mayer, K. U., Diewald, M., Solga, H., Sørensen, A., & Trappe, H. (1995). *Kollektiv und Eigensinn: Lebensverläufe in der DDR und danach [Collective and independence: Life courses in the GDR and after]*. Berlin, Germany: Akademie Verlag.

Klüsener, S., & Goldstein, J. R. (2016). A Long-Standing Demographic East–West Divide in Germany. *Population, Space and Place, 22*(1), 5-22. doi:10.1002/psp.1870

Kreyenfeld, M. (2004). Fertility decisions in the FRG and GDR: An analysis with data from the German Fertility and Family Survey. *Demographic Research, S3*, 275-318. doi:10.4054/DemRes.2004.S3.11

Kreyenfeld, M., Konietzka, D., & Walke, R. (2011). Dynamik und Determinanten nichtehelicher Mutterschaft in Ost- und Westdeutschland [Dynamic and determinants of nonmarital motherhood in Eastern and Western Germany]. In J. Brüderl, L. Castiglioni, & N. Schumann (Eds.), *Partnerschaft, Fertilität und intergenerationale Beziehungen: Ergebnisse der ersten Welle des Beziehungs- und Familienpanels [Partnership, Fertility and Intergenerational Relationships: Findings from the First Wave of the German Family Panel]* (pp. 155-174). Würzburg: Ergon Verlag.

Mayer, K. U. (2004). Whose lives? How history, societies, and institutions define and shape life courses. *Research in Human Development, 1*(3), 161-187. doi:10.1207/s15427617rhd0103_3

Piccarreta, R. (2017). Joint sequence analysis: Association and clustering. *Sociological Methods & Research, 46*(2), 252-287. doi:10.1177/0049124115591013

Rueschemeyer, M. (1988). New family forms in a state socialist society: The German Democratic Republic. *Journal of Family Issues, 9*(3), 354-371. doi:10.1177/019251388009003005

Studer, M. (2013). *WeightedCluster library manual: A practical guide to creating typologies of trajectories in the social sciences with R*. LIVES Working Papers 24. LIVES, Lausanne, Switzerland.

Van Winkle, Z. (2018). Family trajectories across time and space: Increasing complexity in family life courses in Europe? *Demography*, *55*. doi:10.1007/s13524-017-0628-5
